# Short-term PsychoEducation for Carers To Reduce Over Medication of people with intellectual disabilities (SPECTROM): study protocol

**DOI:** 10.1136/bmjopen-2020-037912

**Published:** 2020-04-08

**Authors:** Shoumitro Deb, Bharati Limbu, Mike Crawford, Tim Weaver

**Affiliations:** 1 Division of Psychiatry, Faculty of Medicine, Department of Brain Sciences, Imperial College London, London, UK; 2 Department of Mental Health Research, School of Health and Education, Middlesex University, London, UK

**Keywords:** psychiatry, clinical pharmacology, quality in health care

## Abstract

**Introduction:**

Psychotropic medications that are primarily licenced for the treatment of psychiatric disorders are used widely (32%–85%) among people with intellectual disabilities (ID) often for the management of problem (challenging) behaviour in the absence of a psychiatric disorder. Care staff play a pivotal role in the prescribing process. Currently, no staff training programme exists to address the issue of overprescribing of psychotropic medication in people with ID, thus highlighting an urgent need for developing a psychoeducational programme (PEP) specifically designed to address this issue. We propose to develop a PEP for care staff using the methodology described in the UK Medical Research Council guide for complex interventions.

**Methods and analysis:**

The development of the PEP will involve (1) gathering information on available relevant training programmes, (2) running four focus groups with care staff and other professionals to establish the content and format of the PEP, and (3) organising a co-design event involving all relevant stakeholders to discuss the format of the PEP. A core project team will develop the PEP under guidance from the PEP Development Group which will consist of 10–12 relevant stakeholder representatives. Feedback from selected stakeholders on a draft PEP will allow us to refine the PEP before implementation. The PEP will have web-based modules supplemented by face to face training sessions. When the final draft is ready, we will field test the PEP on six to eight care staff from community care homes for people with ID. After completing the field test, we will run a focus group involving participants in the PEP to get feedback on the PEP.

**Ethics and dissemination:**

Ethics approval for this study was waived by the UK Health Regulatory Authority as the study does not collect any patient related information and only include care staff outside the UK NHS. This will be the first ever such universally freely available PEP supported by training manual and slides.

**Strengths and limitations of this study**

- The study uses a co-production design involving all stakeholders from the outset.
- The study is based on a strong theoretical underpinning.
- Psychoeducational programme is developed according to the UK Medical Research Council guideline.
- A stand-alone package freely available from the internet.
- It was not possible to carry out a randomised controlled trial to assess the effectiveness of the new intervention.

## Introduction

Psychotropic medications are used widely (32%–85%; average 49%–63%) among people with intellectual (learning) disabilities (ID).^1 2^ Many people with ID are also prescribed additional medication for coexisting physical health problems.^3^ Psychotropic medications are used for both to treat mental illness and also used, off licence, for problem (challenging) behaviour such as aggression.^4^ Problem behaviours are more common in people with ID than in the non-ID general population.^5^ In a previous study, Clarke and colleagues found that 36% of those who received psychotropic medication did not have a psychiatric diagnosis.^6^ A recent general practitioner (GP) register based study in the UK has shown that “of 9135 peoples treated with antipsychotic drugs, 6503 (71%) did not have a record of severe mental illness.^2^ Of the 11 915 with a record of challenging behaviour, 5562 (47%) had received antipsychotic drugs. Of those with a record of prescription of antipsychotics, 2362 (26%) did not have a record of severe mental illness or challenging behaviour.” In the UK, the National Institute for Health and Care Excellence (NICE) recommends non-pharmacological intervention in the first instance for the management of problem behaviour in people with ID.^7^

Off-licence use of psychotropic medication in people with ID is a public health concern because of lack of evidence demonstrating their effectiveness in treating problem behaviour in the absence of a diagnosed psychiatric diagnosis,^8^ the high rate of adverse effects which can be difficult to assess^9^ and difficulty in withdrawing people from some medications.^10^ Because of these concerns, NHS England in the UK launched a major initiative called ‘Stopping Over-Medication of People with a Learning Disability, Autism or Both’ (STOMP).^11^ STOMP aims to prevent inappropriate use of medication for people with ID. However, this initiative does not address the important role that staff carers of people with ID might play in efforts to reduce the use of psychotropic medication.

In a previous study, we found only a few carers (both paid and family carers) explicitly reflected on how their own behaviour may influence aggressive behaviour in a person with ID.^12^ Carers felt that they would benefit from information and training on the role that they and the wider environment may play in triggering problem behaviour. Another recent qualitative interview study of ours has shown that many professionals and family carers feel that care staff need more knowledge and training in the use of medication for the management of problem behaviour in people with ID.^13^

A recent review has found that a number of small studies of staff training under ‘proactive’, ‘reactive’ and ‘supportive’ training framework have shown improved management of problem behaviour in people with ID (see table 1).^14^ However, none of these training programmes is specifically designed to reduce overuse of psychotropic medication. A recent Cochrane review found four randomised controlled trials (RCTs) of psychoeducational programme (PEP) directed to care staff to reduce antipsychotic use to manage challenging behaviour in people with dementia.^15^ All studies showed a reduction in antipsychotic use at end point.

**Table 1:** Types of training currently available for staff who support people with intellectual disabilities

Proactive training	Reactive training	Responsive training
Person centred values and attitudes training Increasing knowledge Training on the communicative aspects of challenging behaviour Training on attributions of challenging behaviour Training on emotional intelligence Training on risk assessment/risk management culture	Appropriate physical skills training	Nidotherapy Behavioural skills training Incident analysis Formulation
**Proactive support**	**Reactive support**	**Responsive support**
Reflective practice groups Supervision Stress management processes	Post incident support	Individual support (formal and informal) Psychological support

A properly designed PEP should increase care staff knowledge and understanding of the issues and support a positive attitude towards managing problem behaviour using alternatives to medication. This should subsequently help to reduce overmedication of people with ID. This in turn has the potential to improve the quality of life of people with ID. We, therefore, propose to develop a PEP for paid care staff which will follow the principles laid down in the International Guide for the use of medication to manage challenging behaviour in adults with ID.^16^

### Study aims and objectives

The overall study aim is to undertake pretrial development and testing of a PEP which has the potential to reduce overprescribing of psychotropic medication among people with ID by informing, empowering and equipping care staff with the skills they need to better understand problem behaviour, manage their psychological responses to it and negotiate alternative strategies for reducing problem behaviour without an over reliance on the use of psychotropic medication.

The specific research objectives are as follows:

1. To develop a PEP for care staff in community settings to help reduce the overuse of psychotropic medication among adults with ID.
2. To carry out a field testing of the PEP in preparation for a future RCT.
3. To conduct a process evaluation to gather feedback from participants in the PEP training to assess implementation issues.

## Methods

We will develop the PEP according to Medical Research Council (MRC) guideline for the development and evaluation of complex intervention (see table 2).^17 18^ This will involve a PEP developmental phase using a collaborative co-production approach followed by field testing with embedded process evaluation.

**Table 2:** Framework for the development and evaluation of complex interventions (MRC guide)

Phases	MRC recommended methods	Our proposed method
1. Preclinical/theoretical	Review relevant theory and evidence to ensure (1) best choice of intervention, and (2) predict major confounders and strategic design issues	Collating information from: Systematic review of PEPs in neurodevelopmental disorders (underway) Current e-modules available for treatment in various psychiatric disorders such as schizophrenia, bipolar disorder, anxiety disorder and so on. Outcome of the STOMP initiative, and other e-modules and training programmes for care staff in the field of ID
2. Modelling	Identify (1) intervention components, and (2) how intervention components interrelate and relate to surrogate or final health outcomes	Co-development of PEP with stakeholders (through focus groups and co-design event) to identify the targets for the intervention (and their relation to health outcomes), its components (content) and model(s) of delivery
3(a). Operationalisation	Describe components of a replicable intervention	Formalising of action plans developed in the co-design event by the PEP Development Group to finalise the content and delivery of the PEP
3(b). Piloting exploratory trials	Describe a feasible trial protocol for comparing the intervention to an appropriate alternative	Instead of a full feasibility study we are now proposing an initial field testing and refinement of the PEP
4. Definitive RCT	Compare a fully defined theory-based intervention to an appropriate alternative, using a protocol that is reproducible and adequately controlled in a study with appropriate statistical power	We intend to apply for funding for a future definitive RCT using cluster randomisation methods in community care homes for people with ID with a built-in pilot study
5. Long-term implementation and monitoring	Determine whether the intervention and results can be reliably replicated in uncontrolled settings over the long term	Should be future work after the proposed RCT results become available

ID, intellectual disabilities; MRC, Medical Research Council; PEP, psychoeducational programme; RCT, randomised controlled trial.

### Theoretical framework

We will apply the model of ‘The Theory of Planned behaviour’ (TPB) while developing the PEP.^19^ This theory links one's beliefs with behaviour. According to this theory, human behaviour is guided by three kinds of consideration, ‘behavioural beliefs’ (what the person believes), ‘normative beliefs’ (expected beliefs) and ‘control beliefs’ (outside factors controlling their beliefs). In our study, various aspects of care staff’s beliefs will be explored and the project will try to influence beliefs in order to encourage positive behaviour, which will be measured by assessing care staff’s beliefs, particularly controllability of and attribution to the problem behaviour.

Crucially, behavioural change interventions (eg, training care staff) may only work on improving knowledge, without paying attention to the other factors which limits the potential for change as it is only targeting one area (eg, capacity). Motivation is often overlooked and relates to attitudes/attributes/beliefs. The TPB framework will be integrated both within the development of the training package and the evaluation of the package through Process Evaluation (see later).

### Study setting and participants

The following groups have been identified as stakeholders:

- Adults with ID.
- Their families.
- Care staff working with people with ID and managers of services.
- Community Learning Disability Team members.
- General practitioners and pharmacists.
- Service provider organisations.

We will involve all stakeholders in the development of the PEP. Stakeholders will be identified for participation in all stages of the project through care provider organisations such as Achieve Together (formerly CMG), Dimensions-UK, and Voluntary Organisations Disability Group (VODG) (an umbrella organisation of social care service provider organisations in the UK), Challenging Behaviour Foundation (CBF; a family carer organisation), and project group members’ contacts. The Director of Quality and Clinical Care in Achieve Together (a service provider organisation for people with ID) is also a project group member.

The project will be carried out under three phases.

### Phase I: development of the PEP

Phase I is broken down into the following components;

- Collate information on existing training programme to avoid unnecessary duplication.
- Focus groups to establish stakeholders’ views and preferences on the content and format of the PEP.
- Co-design event to bring together stakeholders to discuss the content and the format of the PEP.
- PEP Development Group meetings.
- Feedback from selected stakeholders on a draft PEP outline.

We will use Experience-based co-design (EBCD) method to develop the PEP.^20^ EBCD is a collaborative approach that aims to improve healthcare services by enabling service-users, carers and staff (ground-level and management) to collaborate together to co-design better services. The approach draws on participatory action research, user-centred design, learning theory and narrative-based approaches to change.^21^ It was first piloted in a Head and Neck Cancer service,^22^ and subsequently a toolkit was developed by the King’s Fund.^23^ Its distinctive features are that it is evidence-based, that it uses people’s *experiences* for that evidence and that it involves close and equal collaboration among all the groups of people who may have a stake in the system or process which is to be improved.

We will set up a PEP Development Group (PEP-DG) (10–12 people) comprising all the stakeholder groups’ representatives mentioned earlier. The group will meet three times. The first meeting will set the scene and decide on the terms of reference. The group will then meet after information gathering on existing training programmes and focus groups to discuss the results and set up the co-design event. In the third meeting, after the co-design event, the outline for the PEP will be confirmed.

A core research team consisting of the principal investigator, the researcher and some key project group members will liaise regularly to develop the PEP components under the PEP-DG’s guidance. We expect the PEP modules to be based on (1) behaviour principles, (2) different behaviour management techniques, (3) role playing/case-based discussion, (4) homework assignments, and (5) review and feedback.^24^ In order to make the intervention replicable, we will develop a manual along with slides, and videos of case vignettes. The manual will clearly describe tasks for each session, trainer’s scripts and other training material such as workbook, activity sheet, videos and homework tasks, as well as data collection form.

The PEP will be developed using a reiterative process of core research group initially producing different modules through regular meetings among them and by collating information from various sources such as the information gathered on existing training programmes, from focus groups and co-design event discussions but also guided by regular feedback received from the PEP-DG to fine-tune the materials.

#### Qualitative work (focus groups)

Qualitative research in phase I of the study will involve focus groups with key stakeholders. An interview schedule will be developed that can be employed flexibly and be open to emergent themes but framed using the Theory of Planned behaviour, thus examining beliefs and attitudes (eg, about psychotropic medication and alternative approaches such as positive behavioural support) and how these might influence behaviour (eg, in terms of requesting support from professionals to prescribe medication or provide help with alternative approaches).

As this is primarily a qualitative research, the sample will be recruited purposively. Voluntary, informed consent will be obtained from all participants before focus group interviews. We will retain the contact details until the end of the study of those who would like to receive a final summary report. Participants will also be invited to take part in the co-design event.

The sample size is a pragmatic decision. No formal sample size calculation is required for this study. This is a small study with limited resources and includes primarily qualitative data collection. The minimum sample size we are aiming for is 8–10 care staff in each focus group (there will be two separate focus groups) and 6–8 care staff for the field testing of the PEP.

We will conduct four focus groups: two with care staff only and two with service managers, trainers/learning heads of service provider organisations and CLDT members. Participants will be purposively sampled for each group to include a range of care staff from different community settings. A researcher with previous experience of conducting qualitative research will run the focus groups under supervision of an expert in qualitative research (TW). We will use the approach used in our previous studies of interviewing carers of people with ID as well as head injury.^25 26^ The first two focus groups involving care staff and the service mangers and other stakeholders respectively will explore issues around medication use, and assessment and management of challenging behaviour. The second two focus groups will explore carers’ and other stakeholders’ views respectively on what should be the content and format of the PEP considering what training is already available and where the gaps are. Focus group discussions will be semistructured using a topic guide, audio recorded and transcribed verbatim.

NVivo computer software will be used to manage data and support analysis.^27^ After familiarisation with the data (reading transcripts), an initial coding frame will be developed built on both a priori research questions and themes developed in the data. This coding frame will be refined as data collection and analysis progress. The framework to the data (indexing) will be applied with the aim of allocating all data to a theme (either already defined or emergent at this point). At the analytical stage, constant comparison will be used to discern patterns and divergences in the data and to support the identification of concepts and categories that enable a comprehensive and detailed response to the research questions.^28^ Triangulation will be used on a selected sample of transcripts to maintain inter-rater reliability of data analysis.

#### Co-design event

A 1-day conference of carers and other stakeholders (about 20–30 people) will be organised to further develop the PEP using EBCD model. At the event, facilitators from the core research team will prepare small groups of four to five people for working together by establishing expectations for the day, identifying group rules, describing available support (quiet spaces, ‘floating’ facilitators) and doing some ‘icebreaking’. The core project team will present the content themes for the PEP and if possible, a short 2 min video of an interview with a person with ID who receives medication for problem behaviour will be presented at the outset. Then each group will work on developing an action plan to address their priority area. The action planning process will follow a clear structure and should produce a plan which will identify: the outline of what should be included in each module, what format this should take, and how to deliver this, and have a clear outline of who will do what by when. Each group will be given specific tasks of planning how best to address issues such as (1) the format of the PEP such as web-based, self-guided learning, group training sessions, modular contents with hyperlinks, passive or active learning and so on; and (2) content and format of face-to-face training sessions. The core research group will then collate the action plans to develop the first draft of the PEP which will be presented to the PEP-DG.

#### Wider stakeholder consultation

An outline of the draft PEP will be circulated (either by post or by email where possible) widely to a varied group of stakeholders for feedback (approximately 20–30 people and organisations). Once the wider stakeholder feedback is received, the PEP will be finalised for field testing. The feedback will be studied by the core research group who will take advice from the PEP-DG on those feedback.

#### Intervention

The PEP will equip care staff with the skills and the support to work with people with ID and health professionals in a proactive way to avoid over-reliance on medication and to seek alternatives for the management of problem behaviour. The intervention will have two components: (1) an educational component comprising web-based information material including video-based case studies, and (2) face-to-face training sessions. We envisage that the e-learning will be developed under the following broad headings; (1) information about medication, their indications and adverse effects; (2) causes and effects of problem behaviours and their assessment; (3) non-pharmacological management strategies for problem behaviour; and (4) advice about work-related stress management. Face-to-face training will provide (1) information-based discussion using the e-learning material and solution focused case studies but also (2) direct support for the care staff such as mindfulness training to reduce stress of carer burden and improve psychological mindedness.

The ultimate format and content (and the method of delivery, eg, number of sessions, group size, frequency, duration of sessions) of the PEP will be established in phase I and after the initial field testing. The web-based information is likely to be modular in nature so that this could apply to carers in different settings and cover medical, psychological and social/environmental causes and management of behaviour. The programme will encourage care staff to use a holistic, person-centred approach to behaviour management, highlighting alternatives to medication and considering functional determinants of behaviour. This will follow the principles laid down in our International Guide^16^ and will align with the UK NICE recommendations for interventions for problem behaviour in people with ID.^7^

### Phase II: field testing

#### Setting and eligibility criteria

For field testing, six to eight care staff will be recruited from different group homes in London through more than one service provider organisations. The PEP will be delivered in the preferred presentation format as determined by the development process in phase I. We have a commitment to deliver face to face training in as few sessions as possible. Based on the feedback from the field testing, we will finalise the optimum number of sessions and the format required for a future definitive RCT.

#### Inclusion criteria

- Care staff working with adults with ID within service provider organisations within a community setting.

#### Exclusion criteria

- Care staff working solely with children and within a hospital setting.

#### Outcome measures

The following carer-related measures will be used prior to the training sessions and at the end.

Two questionnaires will be used to assess care staff’s knowledge of medication use (Knowledge of Psychotropic drugs Questionnaire^29^) and attitude towards management of behaviour (attribution) in general and drug use in particular (Management of Aggression and Violence Attitude Scale-Revised-adapted for ID) (MAVAS-R-ID).^30^ We will assess carer’s quality of life (QoL) using SF-36,^31^ EQ-5D-5L,^32^ mental state using Hospital Anxiety and Depression Scale (HADS),^33^ and Carer Well-being and Support Questionnaire (CWSQ).^34^

#### Data analysis

We will not analyse any carer-related outcome data, but they will be used to assess the feasibility of data collection using these specific measures. Therefore, this study will not involve any statistical data analysis.

### Phase III: process evaluation

We will run a focus group of the participants who took part in the training to explore their experience of participating in the study and changes that could be made to ensure that a subsequent definitive trial was acceptable. We will explore participant’s beliefs about the impact of this intervention (including both positive and negative effects), mechanisms of action and factors that facilitate or hinder its successful delivery. These will be audio recorded and transcribed. Data will be professionally transcribed and subjected to thematic analysis aided by NVivo software.^35^ The same methodology will be used as in phase I qualitative study. We shall collect data from the participants on the PEP’s applicability, acceptability, practicality and relevance using an adapted version of the Feasibility scale devised by Zeilinger and colleagues for carers of adults with ID.^36^

As this is primarily a qualitative research, the sample will be recruited purposively. This will involve recruiting care staff from different organisations, different geographical localities and different care settings. Voluntary, informed consent will be obtained from all participants before focus group interviews. We will retain the contact details until the end of the study of those who would like to receive a final summary report.

### Project management

We will set up a project management group which will meet every 4 months (some meetings will be held through teleconference). Additionally, the core research group will meet every 3–4 weeks and will liaise regularly with the project management group and the PEP-DG. We will set up a combined steering and data management committee including independent experts and carers who will oversee the project governance and data management. They will meet every 7 months and the core research team will report to them. Care staff and people with ID (including advocates) advisory groups will provide regular feedback to the project group.

### Project timetable

Start 01.04.2019; duration 18 months. Set up: 1 month; Development of the PEP (phase I): 11 months; Field testing of the PEP and process evaluation (phase II+III): 3 months; Writing reports, papers, HTA application draft for a future definitive RCT: 3 months. Original estimated study end date was 30 September 2020, which may now be delayed because of COVID-19 pandemic.

## Discussion

Every day in England, psychotropic medication is given inappropriately to up to 35000 people with ID, at an estimated cost to the UK NHS of c.£14 million.^37^ In addition, the quality of life of patients and carers will be impacted by adverse effects, premature mortality, the increased burden on family carers (including loss of income), all of which incur further health and societal costs. Hence, the overmedication of people with ID, particularly the use of psychotropic medications for problem (challenging) behaviour in the absence of a diagnosed psychiatric disorder, is a major public health concern in the UK and worldwide.

Care staff play a pivotal role in influencing these prescribing decisions as they know the person with ID best and are supposed to represent the best interests of the person they support. Our proposal to develop a training programme specifically geared at care staff is relevant to the burden of disease priorities and needs of the UK NHS. NHS England has launched a major initiative to address this concern called ‘Stopping Over-Medication of People with a Learning Disability, Autism or Both’ (STOMP).^11^ However, the STOMP programme has not involved development of any structured training programme for care staff. By addressing this critical training need, our work will complement the STOMP initiative and fill in an important gap in the NHS policy framework.

The PEP will not only address knowledge gap among the care staff but also help to develop a positive attitude towards the management of problem behaviour, particularly exploring staff reaction to problem behaviour and how best to address them by understanding the person behind the behaviour. We will do this by following MRC, UK guideline development recommendation. Therefore, we will set up a PEP-DG involving all stakeholders’ representatives and will involve all stakeholders from the outset using a co-production model. The PEP will have a web-based module complemented by a face to face training session. We will carry out a field testing of the PEP and also conduct a process evaluation to gather feedback from participants in the PEP training to assess implementation issues in preparation for a future RCT.

Our PEP will be first ever universally available package freely available for use anywhere in the world. If useful, similar models could be used for PEPs in relation to other disorders and health issues where reduction of medicine use is desirable such as schizophrenia.

Our PEP will be based on a strong theoretical background as this is designed not only to provide knowledge but more importantly to change care staff attitude to empower them which may be a critical mechanism for helping with the reduction in over-reliance in medication in addressing problem behaviour in people with ID as overmedication of people with ID is contrary to NICE recommendation.^7^ Another strength of our PEP is that this will be case vignette based, so the care staff could relate the training with their day to day practice and implement them. The PEP will be stand alone because it will be accompanied by training manual and designed to be accessible to those who have not attended the accompanying face to face training session. Therefore, anyone anywhere in the world can access the training material and use them without any prior training for themselves. Similarly, the care staff could access the training material directly to help themselves. Although the PEP will be developed keeping primarily care staff in mind, but this PEP could be useful to other stakeholders such as family carers and nurses, occupational therapists, speech and language therapists, pharmacists, GPs and even service providers and commissioners.

We will use a co-production model using the EBCD procedure which involves all the stakeholders from the outset. This will make the PEP both evidence-based, but also consensus-based. This should make the PEP acceptable to care staff for whom the programme is designed. This will also provide good face validity to the intervention.

The efficacy of the PEP will not be assessed in this study, but we intend to design and conduct an appropriately designed RCT to address this question in the future. The outcome measures only assess care staff knowledge and attitude which is at the core of the PEP but it is not within the remit of this project to collect any data related to people with ID who are supported by the care staff who will take part in the project. Also given the resource available, we will be able only to measure short-term outcome related to care staff knowledge and attitude.

## Patient and public involvement

The application has been developed jointly with the key stakeholders, namely people with ID, their family carers and care staff. We have also had input from a GP, a pharmacist, a clinical psychologist and community learning disability team members. Two parents and one director of a provider organisation representing care staff are members of the project team.

A number of family carers provided feedback through the family carer organisation, Challenging Behaviour Foundation (CBF). We have also received a large number of comments from care staff. All the relevant stakeholders will remain fully involved all throughout the project. We will set up two advisory groups, one comprising four care staff and another four adults with ID. These groups will meet every 4 months and will provide feedback and advice throughout the project and co-design topic guides for interviews. The advisory groups will also advise on accessible information leaflets and web-based newsletters for the project.

## Ethics and dissemination

Ethics approval for this study was waived by the UK Health Regulatory Authority as the study does not collect any patient-related information and only include care staff outside the UK NHS. People with ID are involved as stakeholders and will act in an advisory capacity only. Consent to enter the study will be sought from each participant only after a full explanation has been given, an information leaflet offered and time allowed for consideration. Signed participant consent will be obtained. The right of the participant to refuse to participate without giving reasons will be respected. All participants are free to withdraw at any time.

The project will produce a PEP for care staff with accompanying manual and workbooks. Dissemination will be ongoing. A project website at Imperial College London hyperlinked with the Royal College of Psychiatrists (professional organisation), MENCAP (carer organisation), VODG and CBF, will host relevant information. A newsletter will be distributed twice a year to update interested parties on the progress of the project and the findings of each stage. Careful attention will be paid to providing accessible information for people with ID and their carers. Accessible newsletters will be distributed via service user organisations and the final guides and reports will be available in accessible format.

We will run a dissemination workshop at the end of the project involving stakeholders and service provider organisations. This event will be free to attend and open to a wide range of delegates. We will disseminate the findings in VODG (an umbrella organisation for disability service providers in the community) and the Royal College of Psychiatrists’ conferences. A summary report will be produced and sent to a wide range of stakeholders. Papers will be prepared for publication in high-impact peer-reviewed academic journals (including open access journals) as well as professional journals to target a wide audience. Findings of the study will also be presented in local, national and international meetings and conferences. All the documents prepared throughout the project will be made available on the project website and be widely and freely available.

## Supplementary Material

Reviewer comments

Author's manuscript
