# Supplementary material for: Short-term PsychoEducation for Carers To Reduce Over Medication of people with intellectual disabilities (SPECTROM): study protocol
Source: BMJ Open. 2020 Apr 8;10(4):e037912. doi: 10.1136/bmjopen-2020-037912 (PMC7245413; doi:10.1136/bmjopen-2020-037912)
Supplement: Author's manuscript [file bmjopen-2020-037912.draft_revisions.pdf]

BMJ Open

BMJ Open is committed to open peer review. As part of this commitment we make the peer review history of every article we publish publicly available.

When an article is published we post the peer reviewers' comments and the authors' responses online. We also post the versions of the paper that were used during peer review. These are the versions that the peer review comments apply to.

The versions of the paper that follow are the versions that were submitted during the peer review process. They are not the versions of record or the final published versions. They should not be cited or distributed as the published version of this manuscript.

BMJ Open is an open access journal and the full, final, typeset and author-corrected version of record of the manuscript is available on our site with no access controls, subscription charges or pay-per-view fees (http://bmjopen.bmj.com).

If you have any questions on BMJ Open's open peer review process please email info.bmjopen@bmj.com

BMJ Open

Short-term PsychoEducation for Carers To Reduce Over Medication of people with intellectual disability (SPECTROM): study protocol.

Journal:	*BMJ Open*
Manuscript ID	bmjopen-2020-037912
Article Type:	Protocol
Date Submitted by the Author:	21-Feb-2020
Complete List of Authors:	Deb, Shoumitro (Shoumi); Imperial College London, Centre for Psychiatry<br>Limbu, Bharati; Imperial College London, Brain Sciences<br>Crawford, Mike; Imperial College London, Psychological Medicine<br>Weaver, Tim ; Middlesex University
Keywords:	PSYCHIATRY, CLINICAL PHARMACOLOGY, Quality in health care < HEALTH SERVICES ADMINISTRATION & MANAGEMENT

SCHOLARONE™
Manuscripts

I, the Submitting Author has the right to grant and does grant on behalf of all authors of the Work (as defined in the below author licence), an exclusive licence and/or a non-exclusive licence for contributions from authors who are: i) UK Crown employees; ii) where BMJ has agreed a CC-BY licence shall apply, and/or iii) in accordance with the terms applicable for US Federal Government officers or employees acting as part of their official duties; on a worldwide, perpetual, irrevocable, royalty-free basis to BMJ Publishing Group Ltd ("BMJ") its licensees and where the relevant Journal is co-owned by BMJ to the co-owners of the Journal, to publish the Work in this journal and any other BMJ products and to exploit all rights, as set out in our licence.

The Submitting Author accepts and understands that any supply made under these terms is made by BMJ to the Submitting Author unless you are acting as an employee on behalf of your employer or a postgraduate student of an affiliated institution which is paying any applicable article publishing charge ("APC") for Open Access articles. Where the Submitting Author wishes to make the Work available on an Open Access basis (and intends to pay the relevant APC), the terms of reuse of such Open Access shall be governed by a Creative Commons licence – details of these licences and which Creative Commons licence will apply to this Work are set out in our licence referred to above.

Other than as permitted in any relevant BMJ Author's Self Archiving Policies, I confirm this Work has not been accepted for publication elsewhere, is not being considered for publication elsewhere and does not duplicate material already published. I confirm all authors consent to publication of this Work and authorise the granting of this licence.

For peer review only

Short-term PsychoEducation for Carers To Reduce Over Medication of people with intellectual disability (SPECTROM): study protocol.

Shoumitro (Shoumi) Deb,[1] Bharati Limbu,[1] Mike Crawford,[1] Tim Weaver.[2]

[1]Department on Brain Sciences, Imperial College London, UK; [2]Middlesex University, UK.

Correspondence to: Professor Shoumitro (Shoumi) Deb, Imperial College London, UK, Division for Psychiatry, 7th Floor Commonwealth Building, Hammersmith Hospital Campus, Du Cane Road, W12 0NN, London, UK. s.deb@imperial.ac.uk

For peer review only

ABSTRACT

Introduction

Psychotropic medications are used widely (32%-85%) among people with intellectual disabilities (ID) often for the management of problem (challenging) behaviour in the absence of a psychiatric disorder. This practice is against the UK NICE recommendation. Care staff play a pivotal role in the prescribing process. Currently no staff training programme exists to address the issue of overprescribing of psychotropic medication in people with ID, thus highlighting an urgent need for developing a psychoeducational programme (PEP) specifically designed to address this issue. We propose to develop a PEP for care staff using the methodology described in the UK MRC guide for complex interventions.

Methods and Analysis

The development of the PEP will involve (a) gathering information on available relevant training programmes, (b) running four focus groups with care staff and other professionals to establish the content and format of the PEP, and (c) organising a co-design event involving all relevant stakeholders to discuss the format of the PEP. A core project team will develop the PEP under guidance from the PEP Development Group which will consist of 10-12 relevant stakeholder representatives. Feedback from selected stakeholders on a draft PEP will allow us to refine the PEP before implementation. The PEP will have web-based modules supplemented by face to face training sessions. When the final draft is ready, we will field test the PEP on 6-8 care staff from community care homes for people with ID. After completing the

For peer review only - http://bmjopen.bmj.com/site/about/guidelines.xhtml

field test, we will run a focus group involving participants in the PEP to get feedback on the PEP.

Ethics and Dissemination

As the project does not involve any patient and only care staff in the community, no ethics approval is required. This will be the first ever universally freely available PEP supported by training manual and slides.

Trial registration number: Not applicable.

Strengths and limitations of this study

- The study uses a co-production design involving all stakeholders from the outset.
- The study is based on a strong theoretical underpinning.
- PEP is developed according to the UK Medical Research Council (MRC) guideline.
- A stand-alone package freely available from the internet.
- It was not possible to carry out an RCT to assess the effectiveness of the new intervention.

INTRODUCTION

Psychotropic medications are used widely (32%-85%; average 49%-63%) among people with intellectual (learning) disabilities (ID).[1 2] Many people with ID are also prescribed additional medication for co-existing physical health problems.[3] Psychotropic medications are used for both to treat mental illness and also used, off-license, for problem (challenging) behaviour such as aggression.[4] Problem behaviours are more common in people with ID than in the non-ID general population.[5] In a previous study, Clarke and colleagues found that 36% of those who received psychotropic medication did not have a psychiatric diagnosis.[6] A recent GP register based study in the UK has shown that of “9135 peoples treated with antipsychotic drugs, 6503 (71%) did not have a record of severe mental illness.[2] Of the 11,915 with a record of challenging behaviour, 5562 (47%) had received antipsychotic drugs. Of those with a record of prescription of antipsychotics, 2362 (26%) did not have a record of severe mental illness or challenging behaviour.” In the UK, the National Institute for Health and Care Excellence (NICE) recommends non-pharmacological intervention in the first instance for the management of problem behaviour in people with ID.[7]

Off-license use of psychotropic medication in people with ID is a public health concern because of lack of evidence demonstrating their effectiveness in treating problem behaviour in the absence of a diagnosed psychiatric disognosis,[8] the high rate of adverse effects which

For peer review only - http://bmjopen.bmj.com/site/about/guidelines.xhtml

can be difficult to assess[9] and difficulty in withdrawing people from some medications.[10] Because of these concerns, NHS England in the UK launched a major initiative called ‘Stopping Over-Medication of People with a Learning Disability, Autism or Both’ (STOMP).[11] STOMP aims to prevent inappropriate use of medication for people with ID. However, this initiative does not address the important role of staff carers of people with ID might play in efforts to reduce the use of psychotropic medication.

In a previous study, we found only a few carers (both paid and family carers) explicitly reflected on how their behaviour may influence aggressive behaviour in a person with ID.[12] Carers felt that they would benefit from information and training on the role that they and the wider environment may play in triggering problem behaviour. Another recent qualitative interview study of ours has shown that many professionals and family carers feel that care staff need more knowledge and training in the use of medication for the management of problem behaviour in people with ID.[13]

A recent review has found that a number of small studies of staff training under ‘proactive’, ‘reactive’ and ‘supportive’ training framework have shown improved management of problem behaviour in people with ID (see Table 1).[14] However, none of these training programmes is specifically designed to reduce overuse of psychotropic medication. A recent Cochrane review found four RCTs of psychoeducational programme (PEP) directed to care staff to reduce antipsychotic use to manage challenging behaviour in people with dementia.[15] All studies showed a reduction in antipsychotic use at end point.

A properly designed PEP should increase care staff knowledge and understanding of the issues and support a positive attitude towards managing problem behaviour using alternatives to medication. This should subsequently help to reduce overmedication of people with ID. This in turn has the potential to improve the quality of life of people with ID. We, therefore, propose to develop a PEP for paid care staff which will follow the principles laid down in the International Guide for the use of medication to manage challenging behaviour in adults with ID.[16]

Study aims and objectives

The overall study aim is to undertake pre-trial development and testing of a PEP which has the potential to reduce overprescribing of psychotropic medication among people with ID by informing, empowering, and equipping care staff with the skills they need to better understand problem behaviour, manage their psychological responses to it and negotiate alternative strategies for reducing problem behaviour without an over reliance on the use of psychotropic medication.

For peer review only - http://bmjopen.bmj.com/site/about/guidelines.xhtml

The specific research objectives are:

1) To develop a PEP for care staff in community settings to help reduce the overuse of psychotropic medication among adults with ID.
2) To carry out a field testing of the PEP in preparation for a future RCT.
3) To conduct a process evaluation to gather feedback from participants in the PEP training to assess implementation issues.

METHODS

We will develop the PEP according to MRC guideline for the development and evaluation of complex intervention (see Table 2).[17 18] This will involve a PEP developmental phase using a collaborative co-production approach followed by field testing with embedded process evaluation.

Theoretical framework

We will apply the model of 'The Theory of Planned behaviour' (TPB) while developing the PEP.[19] This theory links one's beliefs with behaviour. According to this theory, human behaviour is guided by three kinds of consideration, "behavioural beliefs" (what the person believes), "normative beliefs," (expected beliefs), and "control beliefs" (outside factors controlling their beliefs). In our study various aspects of care staff's beliefs will be explored and the project will try to influence beliefs in order to encourage positive behaviour, which will be measured by assessing care staff's beliefs, particularly controllability of and attribution to the problem behaviour.

Crucially, behavioural change interventions (e.g. training care staff) may only work on improving knowledge, without paying attention to the other factors which limits the potential for change as it is only targeting one area (e.g. capacity). Motivation is often overlooked and relates to attitudes/attributes/beliefs. The TPB framework will be integrated both within the development of the training package and the evaluation of the package through Process Evaluation (see later).

Study setting and participants

The following groups have been identified as stakeholders:

• Adults with ID
• Their families
• Care staff working with people with ID and managers of services
• Community Learning Disability Team members

For peer review only - http://bmjopen.bmj.com/site/about/guidelines.xhtml

• General Practitioners and pharmacists
• Service provider organisations

We will involve all stakeholders in the development of the PEP. Stakeholders will be identified for participation in all stages of the project through care provider organisations such as Achieve Together, Dimensions-UK, and Voluntary Organisations Disability Group (VODG) (an umbrella organisation of social care service provider organisations), Challenging Behaviour Foundation (CBF; a family carer organisation), and project group members' contacts. The Director of Quality and Clinical Care in Achieve Together (a service provider organisation for people with ID) is also a project group member.

The project will be carried out under three phases.

Phase I: development of the PEP

Phase I is broken down into the following components;

a) Collate information on existing training programme to avoid unnecessary duplication.
b) Focus groups to establish stakeholders' views and preferences on the content and format of the PEP.
c) Co-design event to bring together stakeholders to discuss the content and the format of the PEP.
d) PEP development group meetings.
e) Feedback from selected stakeholders on a draft PEP outline.

We will use Experience-based co-design (EBCD) method to develop the PEP.[20] EBCD is a collaborative approach that aims to improve health care services by enabling service-users, carers, and staff (ground-level and management) to collaborate together to co-design better services. The approach draws on participatory action research, user-centred design, learning theory, and narrative-based approaches to change.[21] It was first piloted in a Head and Neck Cancer service,[22] and subsequently a toolkit was developed by the King's Fund.[23] Its distinctive features are that it is evidence-based, that it uses people's *experiences* for that evidence, and that it involves close and equal collaboration among all the groups of people who may have a stake in the system or process which is to be improved.

We will set up a PEP Development Group (10-12 people) comprising all the stakeholder groups mentioned earlier. The group will meet three times. The first meeting will set the scene and decide on the terms of reference. The group will then meet after information

For peer review only - http://bmjopen.bmj.com/site/about/guidelines.xhtml

gathering on existing training programmes and focus groups to discuss the results and set up the co-design event. In the third meeting, after the co-design event, the outline for the PEP will be confirmed.

A core research team consisting of the Principal Investigator, the researcher and some key project group members will liaise regularly to develop the PEP components under the PEP development group's guidance. We expect the PEP modules to be based on (a) behaviour principles, (b) different behaviour management techniques, (c) role playing/ case-based discussion, (d) homework assignments, and (e) review and feedback.[24] In order to make the intervention replicable we will develop a manual along with slides, videos of case vignettes. The manual will clearly describe tasks for each session, trainer's scripts, and other training material such as workbook, activity sheet, videos and homework tasks as well as data collection form.

The PEP will be developed using a re-iterative process of core research group initially producing different modules through regular meetings among them and by collating information from various sources such as the information gathered on existing training programmes, from focus groups and co-design event discussions but also guided by regular feedback received from the PEP development group to fine tune the materials.

Qualitative work (focus groups)

Qualitative research in Phase I of the study will involve focus groups with key stakeholders. An interview schedule will be developed that can be employed flexibly and be open to emergent themes but framed using the Theory of Planned behaviour, thus examining beliefs and attitudes (e.g. about psychotropic medication and alternative approaches such as positive behavioural support) and how these might influence behaviour (e.g. in terms of requesting support from professionals to prescribe medication or provide help with alternative approaches).

As this is primarily a qualitative research, the sample will be recruited purposively. Voluntary, informed consent will be obtained from all participants before focus group interviews. We will retain the contact details until the end of the study of those who would like to receive a final summary report. Participants will also be invited to take part in the co-design event.

The sample size is a pragmatic decision. No formal sample size calculation is required for this study. This is a small study with limited resources and includes primarily qualitative data collection. The minimum sample size we are aiming for is 8-10 care staff in each focus group (there will be two separate focus groups) and 6-8 care staff for the field testing of the PEP.

For peer review only - http://bmjopen.bmj.com/site/about/guidelines.xhtml

We will conduct four focus groups: two with care staff only and two with service managers, learning head of provider organisations, and CLDT members. Participants will be purposively sampled for each group to include a range of care staff from different community settings. A researcher with previous experience of conducting qualitative research will run the focus groups under supervision of an expert in qualitative research (TW). We will use the approach used in our previous studies of interviewing carers of people with ID as well as head injury.[25 26] The first two focus groups involving care staff and the service mangers and other stakeholders respectively will explore issues around medication use, and assessment and management of challenging behaviour. The second two focus groups will explore carers' and other stakeholders' views respectively on what should be the content and format of the PEP considering what training is already available and where the gaps are. Focus group discussions will be semi-structured using a topic guide, audio recorded, and transcribed verbatim.

NVivo computer software will be used to manage data and support analysis.[27] After familiarization with the data (reading transcripts) an initial coding frame will be developed built upon both a priori research questions and themes developed in the data. This coding frame will be refined as data collection and analysis progress. The framework to the data (indexing) will be applied with the aim of allocating all data to a theme (either already defined or emergent at this point). At the analytical stage constant comparison will be used to discern patterns and divergences in the data and to support the identification of concepts and categories that enable a comprehensive and detailed response to the research questions.[28] Triangulation will be used on a selected sample of transcripts to maintain inter-rater reliability of data analysis.

Co-design Event

A one-day conference of carers and other stakeholders (about 20-30 people) will be organised to further develop the PEP using EBCD model. At the event, facilitators from the core research team will prepare small groups of 4-5 people for working together by establishing expectations for the day, identifying group rules, describing available support (quiet spaces, 'floating' facilitators) and doing some 'icebreaking'. The core project team will present the content themes for the PEP and if possible, a short 2 min video of an interview with a person with ID who receives medication for problem behaviour will be presented at the outset. Then each group will work on developing an action plan to address their priority area. The action planning process will follow a clear structure and should produce a plan which will identify: the outline of what should be included in each module, what format this should take, and how to deliver this, and have a clear outline of who will do what by when. Each group will be given specific tasks of planning how best to address issues such as (a) the format of the PEP such as web-

For peer review only - http://bmjopen.bmj.com/site/about/guidelines.xhtml

based, self-guided learning, group training sessions, modular contents with hyperlinks, passive or active learning etc., (b) content and format of face to face training sessions. The core research group will then collate the action plans to develop the first draft of the PEP which will be presented to the PEP development group.

Wider stakeholder consultation

An outline of the draft PEP will be circulated (either by post or by email where possible) widely to a varied group of stakeholders for feedback (approximately 20-30 people and organisations). Once the wider stakeholder feedback is received, the PEP will be finalised for field testing. The feedback will be studied by the core research group who will take advice from the PEP development group on those feedbacks.

Intervention

The PEP will equip care staff with the skills and the support to work with people with ID and health professionals in a proactive way to avoid overreliance on medication and to seek alternatives for the management of problem behaviour. The intervention will have two components; (a) an educational component comprising web-based information material including video-based case studies, and (b) face-to face training sessions. We envisage that the e-learning will be developed under the following broad headings; (a) information about medication, their indications and adverse effects, (b) causes and effects of problem behaviours and their assessment, (c) non-pharmacological management strategies for problem behaviour, and (d) advice about work-related stress management. Face to face training will provide (a) information-based discussion using the e-learning material and solution focused case studies but also (b) direct support for the care staff such as mindfulness training to reduce stress of carer burden and improve psychological mindedness.

The ultimate format and content (and the method of delivery, e.g., number of sessions, group size, frequency, duration of sessions) of the PEP will be established in Phase I and after the initial field testing. The web-based information is likely to be modular in nature so that this could apply to carers in different settings and cover medical, psychological and social/environmental causes and management of behaviour. The programme will encourage care staff to use a holistic, person centred approach to behaviour management, highlighting alternatives to medication and considering functional determinants of behaviour. This will follow the principles laid down in our International Guide[16] and will align with the UK NICE recommendations for interventions for problem behaviour in people with ID.[7]

Phase II: field testing

For peer review only - http://bmjopen.bmj.com/site/about/guidelines.xhtml

Setting and eligibility criteria

For field testing, six to eight care staff will be recruited from different group homes in London through more than one service provider organisations. The PEP will be delivered in the preferred presentation format as determined by the development process in Phase I. We have a commitment to deliver face to face training in as few sessions as possible. Based on the feedback from the field testing we will finalise the optimum number of sessions and the format required for a future definitive RCT.

Inclusion Criteria

- Care staff working with adults with ID within service provider organisations within a community setting.

Exclusion Criteria

- Care staff working solely with children and within a hospital setting.

Outcome measures

The following carer related measures will be used prior to the training sessions and at the end.

Two questionnaires will be used to assess care staff's knowledge of medication use (Knowledge of Psychotropic drugs Questionnaire[29] and attitude towards management of behaviour (attribution) in general and drug use in particular (Management of Aggression and Violence Attitude Scale-Revised-adapted for ID) (MAVAS-R-ID).[30] We will assess carer's quality of life (QoL) using SF-36,[31] EQ-5D-5L,[32] mental state using Hospital Anxiety and Depression Scale (HADS),[33] and Carer Well-being and Support Questionnaire (CWSQ).[34]

Data analysis

We will not analyse any carer related outcome data, but they will be used to assess the feasibility of data collection using these specific measures. Therefore, this study will not involve any statistical data analysis.

Phase III: process evaluation

We will run a focus group of the participants who took part in the training to explore their experience of participating in the study and changes that could be made to ensure that a subsequent definitive trial was acceptable. We will explore participant's beliefs about the

For peer review only - http://bmjopen.bmj.com/site/about/guidelines.xhtml

impact of this intervention (including both positive and negative effects), mechanisms of action, and factors that facilitate or hinder its successful delivery. These will be audio recorded and transcribed. Data will be professionally transcribed and subjected to thematic analysis aided by NVivo software.[35] The same methodology will be used as in Phase I qualitative study. We shall collect data from the participants on the PEP's applicability, acceptability, practicality and relevance using an adapted version of the Feasibility scale devised by Zeilinger and colleagues for carers of adults with ID.[36]

As this is primarily a qualitative research, the sample will be recruited purposively. This will involve recruiting care staff from different organisations, different geographical localities and different care settings. Voluntary, informed consent will be obtained from all participants before focus group interviews. We will retain the contact details until the end of the study of those who would like to receive a final summary report.

Project management

We will set up a project management group which will meet every four months (some meetings will be held through teleconference). Additionally, the core research group will meet every 3-4 weeks and will liaise regularly with the project management group and the PEP development group. We will set up a combined steering and data management group including independent experts and carers who will oversee the project governance and data management. They will meet every 7 months and the core research team will report to them. Care staff and people with ID (including advocates) advisory groups will provide regular feedback to the project group.

Project timetable

Start 01.04.2019; duration 18 months. Set up: 1 month; Development of the PEP (Phase I): 11 months; Field testing of the PEP and process evaluation (Phase II + III): 3 months; Writing reports, papers, HTA application draft for a future definitive RCT: 3 months.

DISCUSSION

Every day in England, psychotropic medication is given inappropriately to up to 35,000 people with ID, at an estimated cost to the UK NHS of c.£14 million.[37] In addition, the quality of life of patients and carers will be impacted by adverse effects, premature mortality, the increased burden on family carers (including loss of income) all of which incur further health and societal costs. Hence, the overmedication of people with ID, particularly the use of psychotropic medications for problem (challenging) behaviour in the absence of a diagnosed psychiatric disorder is a major public health concern in the UK and worldwide.

For peer review only - http://bmjopen.bmj.com/site/about/guidelines.xhtml

Care staff play a pivotal role in influencing these prescribing decisions as they know the person with ID best and are supposed to represent the best interests of the person they support. Our proposal to develop a training programme specifically geared at care staff is relevant to the burden of disease priorities and needs of the UK NHS. NHS England has launched a major initiative to address this concern called ‘Stopping Over-Medication of People with a Learning Disability, Autism or Both’ (STOMP).[11] However, the STOMP programme has not involved development of any structured training programme for care staff. By addressing this critical training need, our work will complement the STOMP initiative and fill in an important gap in the NHS policy framework.

The PEP will not only address knowledge gap among the care staff but also help to develop a positive attitude towards the management of problem behaviour, particularly exploring staff reaction to problem behaviour and how best to address them by understanding the person behind the behaviour. We will do this by following MRC, UK guideline development recommendation. Therefore, we will set up a PEP-Development group involving all stakeholders’ representatives and will involve all stakeholders from the outset using a co-production model. The PEP will have a web-based module complemented by a face to face training session. We will carry out a field testing of the PEP and also conduct a process evaluation to gather feedback from participants in the PEP training to assess implementation issues in preparation for a future RCT.

Our PEP will be first ever universally available package freely available for use anywhere in the world. If useful, similar models could be used for PEPs in relation to other disorders and health issues where reduction of medicine use is desirable such as schizophrenia.

Our PEP will be based on a strong theoretical background as this is designed not only to provide knowledge but more importantly to change care staff attitude to empower them which may be a critical mechanism for helping with the reduction in overreliance in medication in addressing problem behaviour in people with ID as overmedication of people with ID is contrary to the NICE recommendation.[7] Another strength of our PEP is that this will be case vignette based, so the care staff could relate the training with their day to day practice and implement them. The PEP will be stand alone because it will be accompanied by training manual and designed to be accessible to those who have not attended the accompanying face to face training session. Therefore, anyone anywhere in the world can access the training material and use them without any prior training for themselves. Similarly, the care staff could access the training material directly to help themselves. Although the PEP will be developed keeping primarily care staff in mind, but this PEP could be useful to other stakeholders such

For peer review only - http://bmjopen.bmj.com/site/about/guidelines.xhtml

as family carers and nurses, occupational therapists, speech and language therapists, pharmacists, GPs and even service providers and commissioners.

We will use a co-production model using the EBCD procedure which involves all the stakeholders from the outset. This will make the PEP both evidence-based, but also consensus-based. This should make the PEP acceptable to care staff for whom the programme is designed. This will also provide good face validity to the intervention.

The efficacy of the PEP will not be assessed in this study, but we intend to design and conduct an appropriately designed RCT to address this question in the future. The outcome measures only assess care staff knowledge and attitude which is at the core of the PEP but it is not within the remit of this project to collect any data related to people with ID who are supported by the care staff who will take part in the project. Also given the resource available, we will be able only to measure short term outcome related to care staff knowledge and attitude.

PATIENT AND PUBLIC INVOLVEMENT

The application has been developed jointly with the key stakeholders, namely people with ID, their family carers and care staff. We have also had input from a GP, a pharmacist, a clinical psychologist and community learning disability team members. Two parents and one Director of a provider organisation representing care staff are members of the project team.

A number of family carers provided feedback through the family carer organisation, Challenging Behaviour Foundation (CBF). We have also received a large number of comments from care staff. All the relevant stakeholders will remain fully involved all throughout the project. We will set up two advisory groups, one comprising four care staff and another four adults with ID. These groups will meet every 4 months and will provide feedback and advice throughout the project and co-design topic guides for interviews. The advisory groups will also advise on accessible information leaflets and web-based newsletters for the project.

ETHICS AND DISSEMINATION

The project will produce a PEP for care staff with accompanying manual and workbooks. Dissemination will be ongoing. A project website at Imperial College London hyperlinked with the Royal College of Psychiatrists (professional organisation), MENCAP (carer organisation), VODG and CBF, will host relevant information. A newsletter will be distributed twice a year to update interested parties on the progress of the project and the findings of each stage. Careful attention will be paid to providing accessible information for people with ID and their carers. Accessible newsletters will be distributed via service user organisations and the final guides and reports will be available in accessible format.

For peer review only - http://bmjopen.bmj.com/site/about/guidelines.xhtml

We will run a dissemination workshop at the end of the project involving stakeholders and service provider organisations. This event will be free to attend and open to a wide range of delegates. We will disseminate the findings in VODG (an umbrella organisation for disability service providers in the community) and the Royal College of Psychiatrists' conferences. A summary report will be produced and sent to a wide range of stakeholders. Papers will be prepared for publication in high impact peer reviewed academic journals (including open access journals) as well as professional journals to target a wide audience. Findings of the study will also be presented in local, national and international meetings and conferences. All the documents prepared throughout the project will be made available on the project website and be widely and freely available.

Consent to enter the study will be sought from each participant only after a full explanation has been given, an information leaflet offered, and time allowed for consideration. Signed participant consent will be obtained. The right of the participant to refuse to participate without giving reasons will be respected. All participants are free to withdraw at any time.

AUTHOR AFFILIATIONS

[1]Imperial College London, Faculty of Medicine, Department of Brain Sciences, Division of Psychiatry, 7th Floor Commonwealth Building, Hammersmith Hospital Campus, Du Cane Road, London W12 0NN, UK. [2]Department of Mental Health Research, School of Health and Education, Middlesex University, London, UK.

CONTRIBUTORS

SD was involved in the conception and design of the study. All four authors contributed to the study design and preparation of the manuscript.

FUNDING

This article presents independent research funded by the National Institute for Health Research (NIHR) under its Research for Patient Benefit (RfPB) Programme (Grant Reference Number PB-PG-0817-20010). The views expressed are those of the author(s) and not necessarily those of the NHS, the sponsor or the NIHR or the Department of Health, UK.

COMPETING INTEREST.

None declared.

PATIENT CONSENT FOR PUBLICATION.

Not required.

For peer review only - http://bmjopen.bmj.com/site/about/guidelines.xhtml

ETHICS APPROVAL

The study will be conducted in accordance with the recommendations for physicians involved in research on human subjects adopted by the 18th World Medical Assembly, Helsinki 1964 and later revisions. This will be monitored closely by the sponsor (Central and North West London Partnership NHS Foundation Trust, UK). Both the HRA and the Social Care Ethics Committee, UK indicated that as this project involves only non-NHS staff and no patient, their ethical approval will not be required for this project.

PEER REVIEW

Externally peer reviewed by the review team of the funder NIHR, UK.

AVAILABILITY OF SUPPORTING DATA

The Principal Investigator will preserve the confidentiality of participants taking part in the study and fulfil transparency requirements under the General Data Protection Regulation for health and care research. Only the designated trial investigators will have access to the personal data of the participants and to the final dataset. The focus group transcripts will be anonymised, and care staff outcome data will become property of the sponsor (CNWL Partnership NHS Foundation Trust, UK). Regulatory bodies if appropriate may request access to personal data. There may be opportunity to share data anonymously if necessary and appropriate after proper authorisation and approval are obtained.

ACKNOWLEDGEMENT.

The Imperial Biomedical Research Centre Facility which is funded by the National Institute of Health Research, UK provided support for the study. We also acknowledge the help from the co-applicants (Professor Umesh Chauhan, Professor John Rose, Dr Jean O'Hara, Dr Gemma Unwin, Dr David Branford, Mrs Vivien Cooper, Mrs Linda Woodcock and Mr Michael Fullerton) in formulating the protocol.

REFERENCES

1. Deb S. Psychopharmacology. In: Singh NN, ed. Handbook of Evidence-Based Practices in Intellectual and Developmental Disabilities, Evidence-Based Practices in Behavioral Health. Cham, Switzerland: Springer International Publishing 2016:347-381.

For peer review only - http://bmjopen.bmj.com/site/about/guidelines.xhtml

2. Sheehan R, Hassiotis A, Walters K, *et al.* Mental illness, challenging behaviour, and psychotropic drug prescribing in people with intellectual disability: UK population-based cohort study. ***BMJ*** 2015;351:h4326.
3. Deb S, Fraser WI. The use of psychotropic medication in people with learning disability: towards rational prescribing. ***Hum Psychopharmacol*** 1994;9:259-72.
4. Deb S. Psychopharmacology. In: Tsakanikos E, McCarthy J, eds. Handbook of Psychopathology in Intellectual Disability: Research, Practice, and Policy. New York: Springer Science & Business Media 2013:307-324. (Autism and Child Psychopathology Series).
5. Deb S, Unwin GL, Rojahn J, *et al.* Problem behaviours. In: Bertelli MO, Deb S, Munir K, Hassiotis A, Salvador-Carulla L, eds. Textbook of Psychiatry for Intellectual Disability and Autism. Cham, Switzerland & Geneva, Switzerland: Springer International Publishing & World Psychiatric Association 2020. In Press.
6. Clarke DJ, Kelley S, Thinn K, *et al.* Psychotropic drugs and mental retardation: 1. Disabilities and the prescription of drugs for behaviour and for epilepsy in 3 residential settings. ***J Ment Defic Res*** 1990;28:229-33.
7. National Institute for Health and Care Excellence. Challenging behaviour and learning disabilities: prevention and interventions for people with learning disabilities whose behaviour challenges. NICE Guideline 11, 2015. Available: www.nice.org.uk/guidance/ng11 (accessed 14 Feb 2020).
8. Deb S, Bertelli MO, Rossi M. Psychopharmacology. In: Bertelli MO, Deb S, Munir K, Hassiotis A, Salvador-Carulla L, eds. Textbook of Psychiatry for Intellectual Disability and Autism. Cham, Switzerland & Geneva, Switzerland: Springer International Publishing & World Psychiatric Association 2020. In Press.
9. Sheehan R, Horsfall L, Strydom A, Osborn D, Walters K, Hassiotis A. Movement side effects of antipsychotic drugs in adults with and without intellectual disability: UK population-based cohort study. ***BMJ Open*** 2017;7:e017406.
10. Sheehan R, Hassiotis A. Reduction or discontinuation of antipsychotics for challenging behaviour in adults with intellectual disability: a systematic review. ***Lancet Psychiatry*** 2017;4:238-56.
11. Branford D, Gerrard D, Saleem N, Shaw C, Webster A. Stopping over-medication of people with intellectual disability, Autism or both (STOMP) in England part 1-history and background of STOMP. ***Adv Ment Health Intellect Disabil*** 2019;13:31-40.
12. Unwin GL. A longitudinal observational study of aggressive behaviour in adults with intellectual disabilities (PhD thesis). Birmingham, UK: University of Birmingham, 2013. Available: www.etheses.bham.ac.uk (accessed 14 Feb 2020).

For peer review only - http://bmjopen.bmj.com/site/about/guidelines.xhtml

13. Hassiotis A, Kimona K, Moncrieff J, Deb S. “A stakeholder consultation about future research of psychotropic medication use and behaviour support for adults with Intellectual Disabilities who present with behaviours that challenge: Feasibility of future research” NOCLOR, 2016.
14. Rose J, Gallivan, A. Staff training and supervision. In: Matson JL, ed. Handbook of Intellectual Disabilities: Integrating Theory, Research and Practice. LA, USA: Springer 2020. In Press.
15. Richter T, Meyer G, Möhler R, *et al.* Psychosocial interventions for reducing antipsychotic medication in care home residents. Cochrane Database Syst Rev 2012;12 doi:10.1002/14651858.CD008634.pub2.
16. Deb S, Kwok H, Bertelli M, *et al.* International guide to prescribing psychotropic medication for the management of problem behaviours in adults with intellectual disabilities. ***World Psychiatry*** 2009;8:181-186.
17. Craig P, Dieppe P, Macintyre S, *et al.* Developing and evaluating complex interventions: the new Medical Research Council guidance. ***BMJ*** 2008;337:a1655.
18. Medical Research Council. A framework for the development and evaluation of RCTs for complex interventions to improve health. London: MRC, 2000.
19. Ajzen I. The Theory of Planned Behavior. ***Organ Behav Hum Decis Process*** 1991;50:179-211.
20. Robert G. Participatory action research: Using experience- based co-design (EBCD) to improve the quality of health care services. In: Ziebland S, Calabrese J, Coulter A, Locock L, eds. Understanding and using experiences of health and illness. Oxford: Oxford University Press 2013:138-149.
21. Bate P, Robert G. Experience-based design: From redesigning the system around the patient to co-designing services with the patient. ***BMJ Quality and Safety Health Care*** 2006;15:307-310.
22. Bate P, Robert G. Toward a more user-centric OD: Lessons from the field of experience-based design and a case study. ***J Appl Behav Sci*** 2007;43:41-66.
23. The King’s Fund and The Point of Care Foundation. Experience-based co-design toolkit. 2012. Available:https://www.pointofcarefoundation.org.uk/resource/experience-based-co-design-ebcd-toolkit/ (accessed on 14.02.20).
24. Fossey J, Ballard C, Juszczak E, *et al.* Effect of enhanced psychosocial care on antipsychotic use in nursing home residents with severe dementia: cluster randomised trial. ***BMJ*** 2006;332:756-61.

For peer review only - http://bmjopen.bmj.com/site/about/guidelines.xhtml

25. Deb S, Hare M, Prior L. Symptoms of dementia among adults with Down's syndrome: a qualitative study. *J Intellect Disabil Res* 2007;51:726-739.
26. Morris PG, Prior L, Deb S, *et al.* Patients' views on outcome following head injury: a qualitative study. *BMC Fam Pract* 2005;6:1-6.
27. Hesse-Biber S, Leavy P. The Practice of Qualitative research. Thousand Oaks, CA: Sage Publications 2006.
28. Joffe H. Thematic analysis. In: Harper D, Thompson AR, eds. Qualitative methods in mental health and psychotherapy: A guide for students and practitioners. Chichester: John Wiley & Sons 2011:209-23.
29. de Kuijper G, van der Putten AAJ. Knowledge and expectations of direct support professionals towards effects of psychotropic drug use in people with intellectual disabilities. *J Appl Res Intellect Disabil* 2017;30:1-9.
30. Duxbury J, Hahn S, Needham I, *et al*. The management of aggression and violence attitude scale (MAVAS): a cross-national comparative study. *J Adv Nurs* 2008;62:596-606.
31. Brazier JE, Roberts JF, Deverill MD. The estimation of a preference-based measure of health from the SF-36. *J Health Econ* 2002;21:271-92.
32. Herdman M, Gudex C, Lloyd A, *et al*. Development and preliminary testing of the new five-level version of EQ-5D (EQ-5D-5L). *Qual Life Res* 2011;20:1727-36.
33. Zigmond AS, Snaith RP. The Hospital Anxiety and Depression Scale. *Acta Psychiatr Scand* 1983;67:361-370.
34. Quirk A, Smith S, Hamilton S, *et al*. Development of the carer well-being and support questionnaire. *Ment Health Rev J* 2012;17:128-138.
35. Denzin NK, Lincoln YS. The Sage Handbook of Qualitative Research. 4th ed. Los Angeles: Sage Publications 2011.
36. Zeilinger EL, Gärtner C, Janicki MP, *et al.* Practical applications of the NTG-EDSD for screening adults with intellectual disability for dementia: a German-language version feasibility study. *J Intellect Dev Disabil* 2016;41:42-9.
37. Glover G, Williams R, Branford D. Prescribing of psychotropic drugs to people with learning disabilities and/or autism by general practitioners in England. Public Health England, UK, 2015.

Table 1. Types of training currently available for staff who support people with ID[14]

Proactive Training	Reactive Training	Responsive Training

For peer review only - http://bmjopen.bmj.com/site/about/guidelines.xhtml

• Person centered values and attitudes training<br>• Increasing knowledge<br>• Training on the communicative aspects of challenging behavior<br>• Training on attributions of challenging behavior<br>• Training on emotional intelligence<br>• Training on risk assessment/risk management culture	• Appropriate physical skills training	• Nidotherapy<br>• Behavioral skills training<br>• Incident analysis<br>• Formulation
**Proactive Support**	**Reactive Support**	**Responsive Support**
• Reflective practice groups<br>• Supervision<br>• Stress management processes	• Post incident support	• Individual support (formal and informal)<br>• Psychological support

Table 2. Framework for the development and evaluation of complex interventions (MRC guide)[17 18]

Phases	MRC recommended methods	Our proposed method
1. Pre-clinical/theoretical	Review relevant theory and evidence to ensure (i) best choice of intervention, and (ii) predict major confounders and strategic design issues	Collating information from:<br>Systematic review of PEPs in neurodevelopmental disorders (underway and will be completed before the study begins)<br>Current e-modules available for treatment in various psychiatric disorders such as schizophrenia, bipolar disorder, anxiety disorder etc.<br>Outcome of the STOMP initiative, and other e-modules and training programmes for care staff in the field of ID
2. Modelling	Identify (i) intervention components, and (ii) how	Co-development of PEP with stakeholders (through focus groups and co-design event) to

For peer review only - http://bmjopen.bmj.com/site/about/guidelines.xhtml

	intervention components interrelate and relate to surrogate or final health outcomes	identify the targets for the intervention (and their relation to health outcomes), its components (content) and model(s) of delivery
3(a) Operationalization	Describe components of a replicable intervention	Formalising of action plans developed in the co-design event by the PEP Development Group to finalise the content and delivery of the PEP
3(b) Piloting exploratory trials	Describe a feasible trial protocol for comparing the intervention to an appropriate alternative	Instead of a full feasibility study we are now proposing an initial field testing and refinement of the PEP
4. Definitive randomized controlled trial	Compare a fully defined theory-based intervention to an appropriate alternative, using a protocol that is reproducible and adequately controlled in a study with appropriate statistical power	We intend to apply for funding for a future definitive RCT using cluster randomisation methods in community care homes for people with ID with a built-in pilot study
5.Long-term implementation and monitoring	Determine whether the intervention and results can be reliably replicated in uncontrolled settings over the long term	Should be future work after the proposed RCT results become available

For peer review only - http://bmjopen.bmj.com/site/about/guidelines.xhtml

SPIRIT

STANDARD PROTOCOL ITEMS: RECOMMENDATIONS FOR INTERVENTIONAL TRIALS

SPIRIT 2013 Checklist: Recommended items to address in a clinical trial protocol and related documents*

Section/item	Item No	Description	Addressed on page number
**Administrative information**			
Title	1	Descriptive title identifying the study design, population, interventions, and, if applicable, trial acronym	1
Trial registration	2a	Trial identifier and registry name. If not yet registered, name of intended registry	Not applicable
	2b	All items from the World Health Organization Trial Registration Data Set	Not applicable
Protocol version	3	Date and version identifier	Not applicable
Funding	4	Sources and types of financial, material, and other support	9
Roles and responsibilities	5a	Names, affiliations, and roles of protocol contributors	1 & 9
	5b	Name and contact information for the trial sponsor	9
	5c	Role of study sponsor and funders, if any, in study design; collection, management, analysis, and interpretation of data; writing of the report; and the decision to submit the report for publication, including whether they will have ultimate authority over any of these activities	10
	5d	Composition, roles, and responsibilities of the coordinating centre, steering committee, endpoint adjudication committee, data management team, and other individuals or groups overseeing the trial, if applicable (see Item 21a for data monitoring committee)	Not applicable See page 7 for project management structure

For peer review only

Introduction

Background and rationale	6a	Description of research question and justification for undertaking the trial, including summary of relevant studies (published and unpublished) examining benefits and harms for each intervention	2
	6b	Explanation for choice of comparators	Not applicable
Objectives	7	Specific objectives or hypotheses	3
Trial design	8	Description of trial design including type of trial (eg, parallel group, crossover, factorial, single group), allocation ratio, and framework (eg, superiority, equivalence, noninferiority, exploratory)	Not applicable

Methods: Participants, interventions, and outcomes

Study setting	9	Description of study settings (eg, community clinic, academic hospital) and list of countries where data will be collected. Reference to where list of study sites can be obtained	Not applicable
Eligibility criteria	10	Inclusion and exclusion criteria for participants. If applicable, eligibility criteria for study centres and individuals who will perform the interventions (eg, surgeons, psychotherapists)	6
Interventions	11a	Interventions for each group with sufficient detail to allow replication, including how and when they will be administered	Not applicable but see pages 6 for the description of the intervention which will be used in the field trial.
	11b	Criteria for discontinuing or modifying allocated interventions for a given trial participant (eg, drug dose change in response to harms, participant request, or improving/worsening disease)	Not applicable
	11c	Strategies to improve adherence to intervention protocols, and any procedures for monitoring adherence (eg, drug tablet return, laboratory tests)	Not applicable
	11d	Relevant concomitant care and interventions that are permitted or prohibited during the trial	Not applicable

For peer review only - http://bmjopen.bmj.com/site/about/guidelines.xhtml

Outcomes	12	Primary, secondary, and other outcomes, including the specific measurement variable (eg, systolic blood pressure), analysis metric (eg, change from baseline, final value, time to event), method of aggregation (eg, median, proportion), and time point for each outcome. Explanation of the clinical relevance of chosen efficacy and harm outcomes is strongly recommended	Not applicable but see page 6 for care staff related outcome measures
Participant timeline	13	Time schedule of enrolment, interventions (including any run-ins and washouts), assessments, and visits for participants. A schematic diagram is highly recommended (see Figure)	Not applicable but see page 7 for project timeline.
Sample size	14	Estimated number of participants needed to achieve study objectives and how it was determined, including clinical and statistical assumptions supporting any sample size calculations	5
Recruitment	15	Strategies for achieving adequate participant enrolment to reach target sample size	Not applicable but see page 3 for study settings and participants.

Methods: Assignment of interventions (for controlled trials)

Allocation:

Sequence generation	16a	Method of generating the allocation sequence (eg, computer-generated random numbers), and list of any factors for stratification. To reduce predictability of a random sequence, details of any planned restriction (eg, blocking) should be provided in a separate document that is unavailable to those who enrol participants or assign interventions	Not applicable
Allocation concealment mechanism	16b	Mechanism of implementing the allocation sequence (eg, central telephone; sequentially numbered, opaque, sealed envelopes), describing any steps to conceal the sequence until interventions are assigned	Not applicable
Implementation	16c	Who will generate the allocation sequence, who will enrol participants, and who will assign participants to interventions	Not applicable
Blinding (masking)	17a	Who will be blinded after assignment to interventions (eg, trial participants, care providers, outcome assessors, data analysts), and how	Not applicable

	17b	If blinded, circumstances under which unblinding is permissible, and procedure for revealing a participant's allocated intervention during the trial	Not applicable
**Methods: Data collection, management, and analysis**			
Data collection methods	18a	Plans for assessment and collection of outcome, baseline, and other trial data, including any related processes to promote data quality (eg, duplicate measurements, training of assessors) and a description of study instruments (eg, questionnaires, laboratory tests) along with their reliability and validity, if known. Reference to where data collection forms can be found, if not in the protocol	6: Outcome measure
	18b	Plans to promote participant retention and complete follow-up, including list of any outcome data to be collected for participants who discontinue or deviate from intervention protocols	Not applicable
Data management	19	Plans for data entry, coding, security, and storage, including any related processes to promote data quality (eg, double data entry; range checks for data values). Reference to where details of data management procedures can be found, if not in the protocol	5: Data entry and coding via NVivo & 10 for availability of supporting data
Statistical methods	20a	Statistical methods for analysing primary and secondary outcomes. Reference to where other details of the statistical analysis plan can be found, if not in the protocol	Not applicable, see page 6
	20b	Methods for any additional analyses (eg, subgroup and adjusted analyses)	Not applicable
	20c	Definition of analysis population relating to protocol non-adherence (eg, as randomised analysis), and any statistical methods to handle missing data (eg, multiple imputation)	Not applicable
**Methods: Monitoring**			
Data monitoring	21a	Composition of data monitoring committee (DMC); summary of its role and reporting structure; statement of whether it is independent from the sponsor and competing interests; and reference to where further details about its charter can be found, if not in the protocol. Alternatively, an explanation of why a DMC is not needed	Not applicable as SPECTROM is not an RCT but a combined data management and steering committee will be set up, see page 7

	21b	Description of any interim analyses and stopping guidelines, including who will have access to these interim results and make the final decision to terminate the trial	Not applicable
Harms	22	Plans for collecting, assessing, reporting, and managing solicited and spontaneously reported adverse events and other unintended effects of trial interventions or trial conduct	Not applicable
Auditing	23	Frequency and procedures for auditing trial conduct, if any, and whether the process will be independent from investigators and the sponsor	Not applicable
**Ethics and dissemination**			
Research ethics approval	24	Plans for seeking research ethics committee/institutional review board (REC/IRB) approval	Not required as no patient data are collected, see page 9
Protocol amendments	25	Plans for communicating important protocol modifications (eg, changes to eligibility criteria, outcomes, analyses) to relevant parties (eg, investigators, REC/IRBs, trial participants, trial registries, journals, regulators)	Not applicable
Consent or assent	26a	Who will obtain informed consent or assent from potential trial participants or authorised surrogates, and how (see Item 32)	9
	26b	Additional consent provisions for collection and use of participant data and biological specimens in ancillary studies, if applicable	Not applicable
Confidentiality	27	How personal information about potential and enrolled participants will be collected, shared, and maintained in order to protect confidentiality before, during, and after the trial	10
Declaration of interests	28	Financial and other competing interests for principal investigators for the overall trial and each study site	9: None declared
Access to data	29	Statement of who will have access to the final trial dataset, and disclosure of contractual agreements that limit such access for investigators	10
Ancillary and post-trial care	30	Provisions, if any, for ancillary and post-trial care, and for compensation to those who suffer harm from trial participation	Not applicable

For peer review only

Dissemination policy	31a	Plans for investigators and sponsor to communicate trial results to participants, healthcare professionals, the public, and other relevant groups (eg, via publication, reporting in results databases, or other data sharing arrangements), including any publication restrictions	8-9
	31b	Authorship eligibility guidelines and any intended use of professional writers	9
	31c	Plans, if any, for granting public access to the full protocol, participant-level dataset, and statistical code	9-10
**Appendices**			
Informed consent materials	32	Model consent form and other related documentation given to participants and authorised surrogates	yes
Biological specimens	33	Plans for collection, laboratory evaluation, and storage of biological specimens for genetic or molecular analysis in the current trial and for future use in ancillary studies, if applicable	No/ Not applicable

*It is strongly recommended that this checklist be read in conjunction with the SPIRIT 2013 Explanation & Elaboration for important clarification on the items. Amendments to the protocol should be tracked and dated. The SPIRIT checklist is copyrighted by the SPIRIT Group under the Creative Commons "Attribution-NonCommercial-NoDerivs 3.0 Unported" license.

For peer review only

BMJ Open

Short-term PsychoEducation for Carers To Reduce Over Medication of people with intellectual disabilities (SPECTROM): study protocol.

Journal:	*BMJ Open*
Manuscript ID	bmjopen-2020-037912.R1
Article Type:	Protocol
Date Submitted by the Author:	25-Mar-2020
Complete List of Authors:	Deb, Shoumitro (Shoumi); Imperial College London, Centre for Psychiatry<br>Limbu, Bharati; Imperial College London, Brain Sciences<br>Crawford, Mike; Imperial College London, Psychological Medicine<br>Weaver, Tim ; Middlesex University
**Primary Subject Heading**:	Mental health
Secondary Subject Heading:	Mental health
Keywords:	PSYCHIATRY, CLINICAL PHARMACOLOGY, Quality in health care < HEALTH SERVICES ADMINISTRATION & MANAGEMENT

SCHOLARONE™
Manuscripts

I, the Submitting Author has the right to grant and does grant on behalf of all authors of the Work (as defined in the below author licence), an exclusive licence and/or a non-exclusive licence for contributions from authors who are: i) UK Crown employees; ii) where BMJ has agreed a CC-BY licence shall apply, and/or iii) in accordance with the terms applicable for US Federal Government officers or employees acting as part of their official duties; on a worldwide, perpetual, irrevocable, royalty-free basis to BMJ Publishing Group Ltd ("BMJ") its licensees and where the relevant Journal is co-owned by BMJ to the co-owners of the Journal, to publish the Work in this journal and any other BMJ products and to exploit all rights, as set out in our licence.

The Submitting Author accepts and understands that any supply made under these terms is made by BMJ to the Submitting Author unless you are acting as an employee on behalf of your employer or a postgraduate student of an affiliated institution which is paying any applicable article publishing charge ("APC") for Open Access articles. Where the Submitting Author wishes to make the Work available on an Open Access basis (and intends to pay the relevant APC), the terms of reuse of such Open Access shall be governed by a Creative Commons licence – details of these licences and which Creative Commons licence will apply to this Work are set out in our licence referred to above.

Other than as permitted in any relevant BMJ Author's Self Archiving Policies, I confirm this Work has not been accepted for publication elsewhere, is not being considered for publication elsewhere and does not duplicate material already published. I confirm all authors consent to publication of this Work and authorise the granting of this licence.

For peer review only

Short-term PsychoEducation for Carers To Reduce Over Medication of people with intellectual disabilities (SPECTROM): study protocol.

Shoumitro (Shoumi) Deb,[1] Bharati Limbu,[1] Mike Crawford,[1] Tim Weaver.[2]

[1]Department on Brain Sciences, Imperial College London, UK; [2]Middlesex University, UK.

Correspondence to: Professor Shoumitro (Shoumi) Deb, Imperial College London, UK, Division for Psychiatry, 7th Floor Commonwealth Building, Hammersmith Hospital Campus, Du Cane Road, W12 0NN, London, UK. s.deb@imperial.ac.uk

For peer review only

ABSTRACT

Introduction

Psychotropic medications that are primarily licenced for the treatment of psychiatric disorders are used widely (32%-85%) among people with intellectual disabilities (ID) often for the management of problem (challenging) behaviour in the absence of a psychiatric disorder. Care staff play a pivotal role in the prescribing process. Currently no staff training programme exists to address the issue of overprescribing of psychotropic medication in people with ID, thus highlighting an urgent need for developing a psychoeducational programme (PEP) specifically designed to address this issue. We propose to develop a PEP for care staff using the methodology described in the UK MRC guide for complex interventions.

Methods and Analysis

The development of the PEP will involve (a) gathering information on available relevant training programmes, (b) running four focus groups with care staff and other professionals to establish the content and format of the PEP, and (c) organising a co-design event involving all relevant stakeholders to discuss the format of the PEP. A core project team will develop the PEP under guidance from the PEP Development Group which will consist of 10-12 relevant stakeholder representatives. Feedback from selected stakeholders on a draft PEP will allow us to refine the PEP before implementation. The PEP will have web-based modules supplemented by face to face training sessions. When the final draft is ready, we will field test the PEP on 6-8 care staff from community care homes for people with ID. After completing the

For peer review only - http://bmjopen.bmj.com/site/about/guidelines.xhtml

field test, we will run a focus group involving participants in the PEP to get feedback on the PEP.

Ethics and Dissemination

Ethics approval for this study was waived by the UK Health Regulatory Authority as the study does not collect any patient related information and only include care staff outside the UK NHS. This will be the first ever such universally freely available PEP supported by training manual and slides.

Trial registration number: Not applicable.

Strengths and limitations of this study

- The study uses a co-production design involving all stakeholders from the outset.
- The study is based on a strong theoretical underpinning.
- PEP is developed according to the UK Medical Research Council (MRC) guideline.
- A stand-alone package freely available from the internet.
- It was not possible to carry out an RCT to assess the effectiveness of the new intervention.

INTRODUCTION

Psychotropic medications are used widely (32%-85%; average 49%-63%) among people with intellectual (learning) disabilities (ID).[1 2] Many people with ID are also prescribed additional medication for co-existing physical health problems.[3] Psychotropic medications are used for both to treat mental illness and also used, off-license, for problem (challenging) behaviour such as aggression.[4] Problem behaviours are more common in people with ID than in the non-ID general population.[5] In a previous study, Clarke and colleagues found that 36% of those who received psychotropic medication did not have a psychiatric diagnosis.[6] A recent GP register based study in the UK has shown that "of 9135 peoples treated with antipsychotic drugs, 6503 (71%) did not have a record of severe mental illness.[2] Of the 11,915 with a record of challenging behaviour, 5562 (47%) had received antipsychotic drugs. Of those with a record of prescription of antipsychotics, 2362 (26%) did not have a record of severe mental illness or challenging behaviour." In the UK, the National Institute for Health and Care Excellence (NICE) recommends non-pharmacological intervention in the first instance for the management of problem behaviour in people with ID.[7]

Off-license use of psychotropic medication in people with ID is a public health concern because of lack of evidence demonstrating their effectiveness in treating problem behaviour

For peer review only - http://bmjopen.bmj.com/site/about/guidelines.xhtml

in the absence of a diagnosed psychiatric disognosis,[8] the high rate of adverse effects which can be difficult to assess[9] and difficulty in withdrawing people from some medications.[10] Because of these concerns, NHS England in the UK launched a major initiative called ‘Stopping Over-Medication of People with a Learning Disability, Autism or Both’ (STOMP).[11] STOMP aims to prevent inappropriate use of medication for people with ID. However, this initiative does not address the important role that staff carers of people with ID might play in efforts to reduce the use of psychotropic medication.

In a previous study, we found only a few carers (both paid and family carers) explicitly reflected on how their own behaviour may influence aggressive behaviour in a person with ID.[12] Carers felt that they would benefit from information and training on the role that they and the wider environment may play in triggering problem behaviour. Another recent qualitative interview study of ours has shown that many professionals and family carers feel that care staff need more knowledge and training in the use of medication for the management of problem behaviour in people with ID.[13]

A recent review has found that a number of small studies of staff training under ‘proactive’, ‘reactive’ and ‘supportive’ training framework have shown improved management of problem behaviour in people with ID (see Table 1).[14] However, none of these training programmes is specifically designed to reduce overuse of psychotropic medication. A recent Cochrane review found four RCTs of psychoeducational programme (PEP) directed to care staff to reduce antipsychotic use to manage challenging behaviour in people with dementia.[15] All studies showed a reduction in antipsychotic use at end point.

A properly designed PEP should increase care staff knowledge and understanding of the issues and support a positive attitude towards managing problem behaviour using alternatives to medication. This should subsequently help to reduce overmedication of people with ID. This in turn has the potential to improve the quality of life of people with ID. We, therefore, propose to develop a PEP for paid care staff which will follow the principles laid down in the International Guide for the use of medication to manage challenging behaviour in adults with ID.[16]

Study aims and objectives

The overall study aim is to undertake pre-trial development and testing of a PEP which has the potential to reduce overprescribing of psychotropic medication among people with ID by informing, empowering, and equipping care staff with the skills they need to better understand problem behaviour, manage their psychological responses to it and negotiate alternative strategies for reducing problem behaviour without an over reliance on the use of psychotropic medication.

For peer review only - http://bmjopen.bmj.com/site/about/guidelines.xhtml

The specific research objectives are:

1) To develop a PEP for care staff in community settings to help reduce the overuse of psychotropic medication among adults with ID.
2) To carry out a field testing of the PEP in preparation for a future RCT.
3) To conduct a process evaluation to gather feedback from participants in the PEP training to assess implementation issues.

METHODS

We will develop the PEP according to MRC guideline for the development and evaluation of complex intervention (see Table 2).[17 18] This will involve a PEP developmental phase using a collaborative co-production approach followed by field testing with embedded process evaluation.

Theoretical framework

We will apply the model of 'The Theory of Planned behaviour' (TPB) while developing the PEP.[19] This theory links one's beliefs with behaviour. According to this theory, human behaviour is guided by three kinds of consideration, "behavioural beliefs" (what the person believes), "normative beliefs," (expected beliefs), and "control beliefs" (outside factors controlling their beliefs). In our study various aspects of care staff's beliefs will be explored and the project will try to influence beliefs in order to encourage positive behaviour, which will be measured by assessing care staff's beliefs, particularly controllability of and attribution to the problem behaviour.

Crucially, behavioural change interventions (e.g. training care staff) may only work on improving knowledge, without paying attention to the other factors which limits the potential for change as it is only targeting one area (e.g. capacity). Motivation is often overlooked and relates to attitudes/attributes/beliefs. The TPB framework will be integrated both within the development of the training package and the evaluation of the package through Process Evaluation (see later).

Study setting and participants

The following groups have been identified as stakeholders:

• Adults with ID
• Their families
• Care staff working with people with ID and managers of services

For peer review only - http://bmjopen.bmj.com/site/about/guidelines.xhtml

• Community Learning Disability Team members
• General Practitioners and pharmacists
• Service provider organisations

We will involve all stakeholders in the development of the PEP. Stakeholders will be identified for participation in all stages of the project through care provider organisations such as Achieve Together (formerly CMG), Dimensions-UK, and Voluntary Organisations Disability Group (VODG) (an umbrella organisation of social care service provider organisations in the UK), Challenging Behaviour Foundation (CBF; a family carer organisation), and project group members' contacts. The Director of Quality and Clinical Care in Achieve Together (a service provider organisation for people with ID) is also a project group member.

The project will be carried out under three phases.

Phase I: development of the PEP

Phase I is broken down into the following components;

a) Collate information on existing training programme to avoid unnecessary duplication.
b) Focus groups to establish stakeholders' views and preferences on the content and format of the PEP.
c) Co-design event to bring together stakeholders to discuss the content and the format of the PEP.
d) PEP development group meetings.
e) Feedback from selected stakeholders on a draft PEP outline.

We will use Experience-based co-design (EBCD) method to develop the PEP.[20] EBCD is a collaborative approach that aims to improve health care services by enabling service-users, carers, and staff (ground-level and management) to collaborate together to co-design better services. The approach draws on participatory action research, user-centred design, learning theory, and narrative-based approaches to change.[21] It was first piloted in a Head and Neck Cancer service,[22] and subsequently a toolkit was developed by the King's Fund.[23] Its distinctive features are that it is evidence-based, that it uses people's *experiences* for that evidence, and that it involves close and equal collaboration among all the groups of people who may have a stake in the system or process which is to be improved.

We will set up a PEP Development Group (PEP-DG) (10-12 people) comprising all the stakeholder groups' representatives mentioned earlier. The group will meet three times. The

For peer review only - http://bmjopen.bmj.com/site/about/guidelines.xhtml

first meeting will set the scene and decide on the terms of reference. The group will then meet after information gathering on existing training programmes and focus groups to discuss the results and set up the co-design event. In the third meeting, after the co-design event, the outline for the PEP will be confirmed.

A core research team consisting of the Principal Investigator, the researcher and some key project group members will liaise regularly to develop the PEP components under the PEP development group's guidance. We expect the PEP modules to be based on (a) behaviour principles, (b) different behaviour management techniques, (c) role playing/ case-based discussion, (d) homework assignments, and (e) review and feedback.[24] In order to make the intervention replicable we will develop a manual along with slides, and videos of case vignettes. The manual will clearly describe tasks for each session, trainer's scripts, and other training material such as workbook, activity sheet, videos and homework tasks as well as data collection form.

The PEP will be developed using a re-iterative process of core research group initially producing different modules through regular meetings among them and by collating information from various sources such as the information gathered on existing training programmes, from focus groups and co-design event discussions but also guided by regular feedback received from the PEP-DG to fine tune the materials.

Qualitative work (focus groups)

Qualitative research in Phase I of the study will involve focus groups with key stakeholders. An interview schedule will be developed that can be employed flexibly and be open to emergent themes but framed using the Theory of Planned behaviour, thus examining beliefs and attitudes (e.g. about psychotropic medication and alternative approaches such as positive behavioural support) and how these might influence behaviour (e.g. in terms of requesting support from professionals to prescribe medication or provide help with alternative approaches).

As this is primarily a qualitative research, the sample will be recruited purposively. Voluntary, informed consent will be obtained from all participants before focus group interviews. We will retain the contact details until the end of the study of those who would like to receive a final summary report. Participants will also be invited to take part in the co-design event.

The sample size is a pragmatic decision. No formal sample size calculation is required for this study. This is a small study with limited resources and includes primarily qualitative data collection. The minimum sample size we are aiming for is 8-10 care staff in each focus

For peer review only - http://bmjopen.bmj.com/site/about/guidelines.xhtml

group (there will be two separate focus groups) and 6-8 care staff for the field testing of the PEP.

We will conduct four focus groups: two with care staff only and two with service managers, trainers/ learning heads of service provider organisations, and CLDT members. Participants will be purposively sampled for each group to include a range of care staff from different community settings. A researcher with previous experience of conducting qualitative research will run the focus groups under supervision of an expert in qualitative research (TW). We will use the approach used in our previous studies of interviewing carers of people with ID as well as head injury.[25 26] The first two focus groups involving care staff and the service mangers and other stakeholders respectively will explore issues around medication use, and assessment and management of challenging behaviour. The second two focus groups will explore carers' and other stakeholders' views respectively on what should be the content and format of the PEP considering what training is already available and where the gaps are. Focus group discussions will be semi-structured using a topic guide, audio recorded, and transcribed verbatim.

NVivo computer software will be used to manage data and support analysis.[27] After familiarization with the data (reading transcripts) an initial coding frame will be developed built upon both a priori research questions and themes developed in the data. This coding frame will be refined as data collection and analysis progress. The framework to the data (indexing) will be applied with the aim of allocating all data to a theme (either already defined or emergent at this point). At the analytical stage constant comparison will be used to discern patterns and divergences in the data and to support the identification of concepts and categories that enable a comprehensive and detailed response to the research questions.[28] Triangulation will be used on a selected sample of transcripts to maintain inter-rater reliability of data analysis.

Co-design Event

A one-day conference of carers and other stakeholders (about 20-30 people) will be organised to further develop the PEP using EBCD model. At the event, facilitators from the core research team will prepare small groups of 4-5 people for working together by establishing expectations for the day, identifying group rules, describing available support (quiet spaces, 'floating' facilitators) and doing some 'icebreaking'. The core project team will present the content themes for the PEP and if possible, a short 2 min video of an interview with a person with ID who receives medication for problem behaviour will be presented at the outset. Then each group will work on developing an action plan to address their priority area. The action planning process will follow a clear structure and should produce a plan which will identify: the outline of what should be included in each module, what format this should take, and how to deliver

For peer review only - http://bmjopen.bmj.com/site/about/guidelines.xhtml

this, and have a clear outline of who will do what by when. Each group will be given specific tasks of planning how best to address issues such as (a) the format of the PEP such as web-based, self-guided learning, group training sessions, modular contents with hyperlinks, passive or active learning etc., (b) content and format of face to face training sessions. The core research group will then collate the action plans to develop the first draft of the PEP which will be presented to the PEP-DG.

Wider stakeholder consultation

An outline of the draft PEP will be circulated (either by post or by email where possible) widely to a varied group of stakeholders for feedback (approximately 20-30 people and organisations). Once the wider stakeholder feedback is received, the PEP will be finalised for field testing. The feedback will be studied by the core research group who will take advice from the PEP-DG on those feedbacks.

Intervention

The PEP will equip care staff with the skills and the support to work with people with ID and health professionals in a proactive way to avoid overreliance on medication and to seek alternatives for the management of problem behaviour. The intervention will have two components; (a) an educational component comprising web-based information material including video-based case studies, and (b) face-to face training sessions. We envisage that the e-learning will be developed under the following broad headings; (a) information about medication, their indications and adverse effects, (b) causes and effects of problem behaviours and their assessment, (c) non-pharmacological management strategies for problem behaviour, and (d) advice about work-related stress management. Face to face training will provide (a) information-based discussion using the e-learning material and solution focused case studies but also (b) direct support for the care staff such as mindfulness training to reduce stress of carer burden and improve psychological mindedness.

The ultimate format and content (and the method of delivery, e.g., number of sessions, group size, frequency, duration of sessions) of the PEP will be established in Phase I and after the initial field testing. The web-based information is likely to be modular in nature so that this could apply to carers in different settings and cover medical, psychological and social/environmental causes and management of behaviour. The programme will encourage care staff to use a holistic, person centred approach to behaviour management, highlighting alternatives to medication and considering functional determinants of behaviour. This will follow the principles laid down in our International Guide[16] and will align with the UK NICE recommendations for interventions for problem behaviour in people with ID.[7]

For peer review only - http://bmjopen.bmj.com/site/about/guidelines.xhtml

Phase II: field testing

Setting and eligibility criteria

For field testing, six to eight care staff will be recruited from different group homes in London through more than one service provider organisations. The PEP will be delivered in the preferred presentation format as determined by the development process in Phase I. We have a commitment to deliver face to face training in as few sessions as possible. Based on the feedback from the field testing we will finalise the optimum number of sessions and the format required for a future definitive RCT.

Inclusion Criteria

- Care staff working with adults with ID within service provider organisations within a community setting.

Exclusion Criteria

- Care staff working solely with children and within a hospital setting.

Outcome measures

The following carer related measures will be used prior to the training sessions and at the end.

Two questionnaires will be used to assess care staff's knowledge of medication use (Knowledge of Psychotropic drugs Questionnaire[29] and attitude towards management of behaviour (attribution) in general and drug use in particular (Management of Aggression and Violence Attitude Scale-Revised-adapted for ID) (MAVAS-R-ID).[30] We will assess carer's quality of life (QoL) using SF-36,[31] EQ-5D-5L,[32] mental state using Hospital Anxiety and Depression Scale (HADS),[33] and Carer Well-being and Support Questionnaire (CWSQ).[34]

Data analysis

We will not analyse any carer related outcome data, but they will be used to assess the feasibility of data collection using these specific measures. Therefore, this study will not involve any statistical data analysis.

Phase III: process evaluation

For peer review only - http://bmjopen.bmj.com/site/about/guidelines.xhtml

We will run a focus group of the participants who took part in the training to explore their experience of participating in the study and changes that could be made to ensure that a subsequent definitive trial was acceptable. We will explore participant's beliefs about the impact of this intervention (including both positive and negative effects), mechanisms of action, and factors that facilitate or hinder its successful delivery. These will be audio recorded and transcribed. Data will be professionally transcribed and subjected to thematic analysis aided by NVivo software.[35] The same methodology will be used as in Phase I qualitative study. We shall collect data from the participants on the PEP's applicability, acceptability, practicality and relevance using an adapted version of the Feasibility scale devised by Zeilinger and colleagues for carers of adults with ID.[36]

As this is primarily a qualitative research, the sample will be recruited purposively. This will involve recruiting care staff from different organisations, different geographical localities and different care settings. Voluntary, informed consent will be obtained from all participants before focus group interviews. We will retain the contact details until the end of the study of those who would like to receive a final summary report.

Project management

We will set up a project management group which will meet every four months (some meetings will be held through teleconference). Additionally, the core research group will meet every 3-4 weeks and will liaise regularly with the project management group and the PEP development group. We will set up a combined steering and data management committee including independent experts and carers who will oversee the project governance and data management. They will meet every 7 months and the core research team will report to them. Care staff and people with ID (including advocates) advisory groups will provide regular feedback to the project group.

Project timetable

Start 01.04.2019; duration 18 months. Set up: 1 month; Development of the PEP (Phase I): 11 months; Field testing of the PEP and process evaluation (Phase II + III): 3 months; Writing reports, papers, HTA application draft for a future definitive RCT: 3 months. Original estimated study end date was 30th September 2020, which may now be delayed because of Covid 19 pandemic.

DISCUSSION

Every day in England, psychotropic medication is given inappropriately to up to 35,000 people with ID, at an estimated cost to the UK NHS of c.£14 million.[37] In addition, the quality of life of

For peer review only - http://bmjopen.bmj.com/site/about/guidelines.xhtml

patients and carers will be impacted by adverse effects, premature mortality, the increased burden on family carers (including loss of income) all of which incur further health and societal costs. Hence, the overmedication of people with ID, particularly the use of psychotropic medications for problem (challenging) behaviour in the absence of a diagnosed psychiatric disorder is a major public health concern in the UK and worldwide.

Care staff play a pivotal role in influencing these prescribing decisions as they know the person with ID best and are supposed to represent the best interests of the person they support. Our proposal to develop a training programme specifically geared at care staff is relevant to the burden of disease priorities and needs of the UK NHS. NHS England has launched a major initiative to address this concern called ‘Stopping Over-Medication of People with a Learning Disability, Autism or Both’ (STOMP).[11] However, the STOMP programme has not involved development of any structured training programme for care staff. By addressing this critical training need, our work will complement the STOMP initiative and fill in an important gap in the NHS policy framework.

The PEP will not only address knowledge gap among the care staff but also help to develop a positive attitude towards the management of problem behaviour, particularly exploring staff reaction to problem behaviour and how best to address them by understanding the person behind the behaviour. We will do this by following MRC, UK guideline development recommendation. Therefore, we will set up a PEP-Development group involving all stakeholders’ representatives and will involve all stakeholders from the outset using a co-production model. The PEP will have a web-based module complemented by a face to face training session. We will carry out a field testing of the PEP and also conduct a process evaluation to gather feedback from participants in the PEP training to assess implementation issues in preparation for a future RCT.

Our PEP will be first ever universally available package freely available for use anywhere in the world. If useful, similar models could be used for PEPs in relation to other disorders and health issues where reduction of medicine use is desirable such as schizophrenia.

Our PEP will be based on a strong theoretical background as this is designed not only to provide knowledge but more importantly to change care staff attitude to empower them which may be a critical mechanism for helping with the reduction in overreliance in medication in addressing problem behaviour in people with ID as overmedication of people with ID is contrary to the NICE recommendation.[7] Another strength of our PEP is that this will be case vignette based, so the care staff could relate the training with their day to day practice and implement them. The PEP will be stand alone because it will be accompanied by training manual and designed to be accessible to those who have not attended the accompanying

For peer review only - http://bmjopen.bmj.com/site/about/guidelines.xhtml

face to face training session. Therefore, anyone anywhere in the world can access the training material and use them without any prior training for themselves. Similarly, the care staff could access the training material directly to help themselves. Although the PEP will be developed keeping primarily care staff in mind, but this PEP could be useful to other stakeholders such as family carers and nurses, occupational therapists, speech and language therapists, pharmacists, GPs and even service providers and commissioners.

We will use a co-production model using the EBCD procedure which involves all the stakeholders from the outset. This will make the PEP both evidence-based, but also consensus-based. This should make the PEP acceptable to care staff for whom the programme is designed. This will also provide good face validity to the intervention.

The efficacy of the PEP will not be assessed in this study, but we intend to design and conduct an appropriately designed RCT to address this question in the future. The outcome measures only assess care staff knowledge and attitude which is at the core of the PEP but it is not within the remit of this project to collect any data related to people with ID who are supported by the care staff who will take part in the project. Also given the resource available, we will be able only to measure short term outcome related to care staff knowledge and attitude.

PATIENT AND PUBLIC INVOLVEMENT

The application has been developed jointly with the key stakeholders, namely people with ID, their family carers and care staff. We have also had input from a GP, a pharmacist, a clinical psychologist and community learning disability team members. Two parents and one Director of a provider organisation representing care staff are members of the project team.

A number of family carers provided feedback through the family carer organisation, Challenging Behaviour Foundation (CBF). We have also received a large number of comments from care staff. All the relevant stakeholders will remain fully involved all throughout the project. We will set up two advisory groups, one comprising four care staff and another four adults with ID. These groups will meet every 4 months and will provide feedback and advice throughout the project and co-design topic guides for interviews. The advisory groups will also advise on accessible information leaflets and web-based newsletters for the project.

ETHICS AND DISSEMINATION

Ethics approval for this study was waived by the UK Health Regulatory Authority as the study does not collect any patient related information and only include care staff outside the UK NHS. People with ID are involved as stakeholders and will act in an advisory capacity only. Consent to enter the study will be sought from each participant only after a full explanation

For peer review only - http://bmjopen.bmj.com/site/about/guidelines.xhtml

has been given, an information leaflet offered, and time allowed for consideration. Signed participant consent will be obtained. The right of the participant to refuse to participate without giving reasons will be respected. All participants are free to withdraw at any time.

The project will produce a PEP for care staff with accompanying manual and workbooks. Dissemination will be ongoing. A project website at Imperial College London hyperlinked with the Royal College of Psychiatrists (professional organisation), MENCAP (carer organisation), VODG and CBF, will host relevant information. A newsletter will be distributed twice a year to update interested parties on the progress of the project and the findings of each stage. Careful attention will be paid to providing accessible information for people with ID and their carers. Accessible newsletters will be distributed via service user organisations and the final guides and reports will be available in accessible format.

We will run a dissemination workshop at the end of the project involving stakeholders and service provider organisations. This event will be free to attend and open to a wide range of delegates. We will disseminate the findings in VODG (an umbrella organisation for disability service providers in the community) and the Royal College of Psychiatrists' conferences. A summary report will be produced and sent to a wide range of stakeholders. Papers will be prepared for publication in high impact peer reviewed academic journals (including open access journals) as well as professional journals to target a wide audience. Findings of the study will also be presented in local, national and international meetings and conferences. All the documents prepared throughout the project will be made available on the project website and be widely and freely available.

AUTHOR AFFILIATIONS

[1]Imperial College London, Faculty of Medicine, Department of Brain Sciences, Division of Psychiatry, 7th Floor Commonwealth Building, Hammersmith Hospital Campus, Du Cane Road, London W12 0NN, UK. [2]Department of Mental Health Research, School of Health and Education, Middlesex University, London, UK.

CONTRIBUTORS

SD was involved in the conception and design of the study. SD was the lead applicant and the Chief Investigator. All four authors contributed substantially to the study design and preparation of the manuscript and approved the final version of the manuscript. All authors are accountable for all aspects of study design. MC contributed specifically to the MRC complex intervention design. TW contributed specifically to the qualitative research part of the design. BL contributed specifically to the participant information, consent and recruitment part of the design.

For peer review only - http://bmjopen.bmj.com/site/about/guidelines.xhtml

For peer review only

FUNDING

This article presents independent research funded by the National Institute for Health Research (NIHR), UK under its Research for Patient Benefit (RfPB) Programme (Grant Reference Number PB-PG-0817-20010). The views expressed are those of the author(s) and not necessarily those of the NHS, the sponsor or the NIHR or the Department of Health, UK.

COMPETING INTEREST.

None declared.

PATIENT CONSENT FOR PUBLICATION.

Not required.

ETHICS APPROVAL

Ethics approval for this study was waived by the UK Health Regulatory Authority and the Social Care Ethics as the study does not collect any patient related information and only include care staff outside the UK NHS. The study will be conducted in accordance with the recommendations for physicians involved in research on human subjects adopted by the 18th World Medical Assembly, Helsinki 1964 and later revisions. This will be monitored closely by the sponsor (Central and North West London Partnership NHS Foundation Trust, UK).

PEER REVIEW

Externally peer reviewed by the review team of the funder NIHR, UK.

AVAILABILITY OF SUPPORTING DATA

The Principal Investigator will preserve the confidentiality of participants taking part in the study and fulfil transparency requirements under the General Data Protection Regulation for health and care research. Only the designated trial investigators will have access to the personal data of the participants and to the final dataset. The focus group transcripts will be anonymised, and care staff outcome data will become property of the sponsor (CNWL Partnership NHS Foundation Trust, UK). Regulatory bodies if appropriate may request access to personal data. There may be opportunity to share data anonymously if necessary and appropriate after proper authorisation and approval are obtained.

ACKNOWLEDGEMENT.

For peer review only

The Imperial Biomedical Research Centre Facility which is funded by the National Institute of Health Research, UK provided support for the study. We also acknowledge the help from the co-applicants (Professor Umesh Chauhan, Professor John Rose, Dr Jean O'Hara, Dr Gemma Unwin, Dr David Branford, Mrs Vivien Cooper, Mrs Linda Woodcock and Mr Michael Fullerton) in formulating the protocol.

REFERENCES

1. Deb S. Psychopharmacology. In: Singh NN, ed. Handbook of Evidence-Based Practices in Intellectual and Developmental Disabilities, Evidence-Based Practices in Behavioral Health. Cham, Switzerland: Springer International Publishing 2016:347-381.
2. Sheehan R, Hassiotis A, Walters K, *et al.* Mental illness, challenging behaviour, and psychotropic drug prescribing in people with intellectual disability: UK population-based cohort study. ***BMJ*** 2015;351:h4326.
3. Deb S, Fraser WI. The use of psychotropic medication in people with learning disability: towards rational prescribing. ***Hum Psychopharmacol*** 1994;9:259-72.
4. Deb S. Psychopharmacology. In: Tsakanikos E, McCarthy J, eds. Handbook of Psychopathology in Intellectual Disability: Research, Practice, and Policy. New York: Springer Science & Business Media 2013:307-324. (Autism and Child Psychopathology Series).
5. Deb S, Unwin GL, Rojahn J, *et al.* Problem behaviours. In: Bertelli MO, Deb S, Munir K, Hassiotis A, Salvador-Carulla L, eds. Textbook of Psychiatry for Intellectual Disability and Autism. Cham, Switzerland & Geneva, Switzerland: Springer International Publishing & World Psychiatric Association 2020. In Press.
6. Clarke DJ, Kelley S, Thinn K, *et al.* Psychotropic drugs and mental retardation: 1. Disabilities and the prescription of drugs for behaviour and for epilepsy in 3 residential settings. ***J Ment Defic Res*** 1990;28:229-33.
7. National Institute for Health and Care Excellence. Challenging behaviour and learning disabilities: prevention and interventions for people with learning disabilities whose behaviour challenges. NICE Guideline 11, 2015. Available: www.nice.org.uk/guidance/ng11 (accessed 14 Feb 2020).
8. Deb S, Bertelli MO, Rossi M. Psychopharmacology. In: Bertelli MO, Deb S, Munir K, Hassiotis A, Salvador-Carulla L, eds. Textbook of Psychiatry for Intellectual Disability and Autism. Cham, Switzerland & Geneva, Switzerland: Springer International Publishing & World Psychiatric Association 2020. In Press.

For peer review only - http://bmjopen.bmj.com/site/about/guidelines.xhtml

9. Sheehan R, Horsfall L, Strydom A, Osborn D, Walters K, Hassiotis A. Movement side effects of antipsychotic drugs in adults with and without intellectual disability: UK population-based cohort study. *BMJ Open* 2017;7:e017406.
10. Sheehan R, Hassiotis A. Reduction or discontinuation of antipsychotics for challenging behaviour in adults with intellectual disability: a systematic review. *Lancet Psychiatry* 2017;4:238-56.
11. Branford D, Gerrard D, Saleem N, Shaw C, Webster A. Stopping over-medication of people with intellectual disability, Autism or both (STOMP) in England part 1-history and background of STOMP. *Adv Ment Health Intellect Disabil* 2019;13:31-40.
12. Unwin GL. A longitudinal observational study of aggressive behaviour in adults with intellectual disabilities (PhD thesis). Birmingham, UK: University of Birmingham, 2013. Available: www.etheses.bham.ac.uk (accessed 14 Feb 2020).
13. Hassiotis A, Kimona K, Moncrieff J, Deb S. "A stakeholder consultation about future research of psychotropic medication use and behaviour support for adults with Intellectual Disabilities who present with behaviours that challenge: Feasibility of future research" NOCLOR, 2016.
14. Rose J, Gallivan, A. Staff training and supervision. In: Matson JL, ed. Handbook of Intellectual Disabilities: Integrating Theory, Research and Practice. LA, USA: Springer 2019. Pp. 153-168.
15. Richter T, Meyer G, Möhler R, *et al.* Psychosocial interventions for reducing antipsychotic medication in care home residents. Cochrane Database Syst Rev 2012;12 doi:10.1002/14651858.CD008634.pub2.
16. Deb S, Kwok H, Bertelli M, *et al.* International guide to prescribing psychotropic medication for the management of problem behaviours in adults with intellectual disabilities. *World Psychiatry* 2009;8:181-186.
17. Craig P, Dieppe P, Macintyre S, *et al.* Developing and evaluating complex interventions: the new Medical Research Council guidance. *BMJ* 2008;337:a1655.
18. Medical Research Council. A framework for the development and evaluation of RCTs for complex interventions to improve health. London: MRC, 2000.
19. Ajzen I. The Theory of Planned Behavior. *Organ Behav Hum Decis Process* 1991;50:179-211.
20. Robert G. Participatory action research: Using experience- based co-design (EBCD) to improve the quality of health care services. In: Ziebland S, Calabrese J, Coulter A, Locock L, eds. Understanding and using experiences of health and illness. Oxford: Oxford University Press 2013:138-149.

For peer review only - http://bmjopen.bmj.com/site/about/guidelines.xhtml

21. Bate P, Robert G. Experience-based design: From redesigning the system around the patient to co-designing services with the patient. ***BMJ Quality and Safety Health Care*** 2006;15:307-310.
22. Bate P, Robert G. Toward a more user-centric OD: Lessons from the field of experience-based design and a case study. ***J Appl Behav Sci*** 2007;43:41-66.
23. The King's Fund and The Point of Care Foundation. Experience-based co-design toolkit. 2012. Available:https://www.pointofcarefoundation.org.uk/resource/experience-based-co-design-ebcd-toolkit/ (accessed on 14.02.20).
24. Fossey J, Ballard C, Juszczak E, *et al*. Effect of enhanced psychosocial care on antipsychotic use in nursing home residents with severe dementia: cluster randomised trial. ***BMJ*** 2006;332:756-61.
25. Deb S, Hare M, Prior L. Symptoms of dementia among adults with Down's syndrome: a qualitative study. ***J Intellect Disabil Res*** 2007;51:726-739.
26. Morris PG, Prior L, Deb S, *et al*. Patients' views on outcome following head injury: a qualitative study. ***BMC Fam Pract*** 2005;6:1-6.
27. Hesse-Biber S, Leavy P. The Practice of Qualitative research. Thousand Oaks, CA: Sage Publications 2006.
28. Joffe H. Thematic analysis. In: Harper D, Thompson AR, eds. Qualitative methods in mental health and psychotherapy: A guide for students and practitioners. Chichester: John Wiley & Sons 2011:209-23.
29. de Kuijper G, van der Putten AAJ. Knowledge and expectations of direct support professionals towards effects of psychotropic drug use in people with intellectual disabilities. ***J Appl Res Intellect Disabil*** 2017;30:1-9.
30. Duxbury J, Hahn S, Needham I, *et al*. The management of aggression and violence attitude scale (MAVAS): a cross-national comparative study. ***J Adv Nurs*** 2008;62:596-606.
31. Brazier JE, Roberts JF, Deverill MD. The estimation of a preference-based measure of health from the SF-36. ***J Health Econ*** 2002;21:271-92.
32. Herdman M, Gudex C, Lloyd A, *et al*. Development and preliminary testing of the new five-level version of EQ-5D (EQ-5D-5L). ***Qual Life Res*** 2011;20:1727-36.
33. Zigmond AS, Snaith RP. The Hospital Anxiety and Depression Scale. ***Acta Psychiatr Scand*** 1983;67:361-370.
34. Quirk A, Smith S, Hamilton S, *et al*. Development of the carer well-being and support questionnaire. ***Ment Health Rev J*** 2012;17:128-138.

For peer review only - http://bmjopen.bmj.com/site/about/guidelines.xhtml

35. Denzin NK, Lincoln YS. The Sage Handbook of Qualitative Research. 4th ed. Los Angeles: Sage Publications 2011.

36. Zeilinger EL, Gärtner C, Janicki MP, *et al.* Practical applications of the NTG-EDSD for screening adults with intellectual disability for dementia: a German-language version feasibility study. *J Intellect Dev Disabil* 2016;41:42-9.

37. Glover G, Williams R, Branford D. Prescribing of psychotropic drugs to people with learning disabilities and/or autism by general practitioners in England. Public Health England, UK, 2015.

Table 1. Types of training currently available for staff who support people with ID

Proactive Training	Reactive Training	Responsive Training
• Person centered values and attitudes training<br>• Increasing knowledge<br>• Training on the communicative aspects of challenging behavior<br>• Training on attributions of challenging behavior<br>• Training on emotional intelligence<br>• Training on risk assessment/risk management culture	• Appropriate physical skills training	• Nidotherapy<br>• Behavioral skills training<br>• Incident analysis<br>• Formulation
**Proactive Support**	**Reactive Support**	**Responsive Support**
• Reflective practice groups<br>• Supervision<br>• Stress management processes	• Post incident support	• Individual support (formal and informal)<br>• Psychological support

Table 2. Framework for the development and evaluation of complex interventions (MRC guide)

Phases	MRC recommended methods	Our proposed method
1. Pre-clinical/theoretical	Review relevant theory and evidence to ensure (i) best choice of intervention, and (ii)	Collating information from:<br>Systematic review of PEPs in neurodevelopmental disorders (underway)

For peer review only - http://bmjopen.bmj.com/site/about/guidelines.xhtml

	predict major confounders and strategic design issues	Current e-modules available for treatment in various psychiatric disorders such as schizophrenia, bipolar disorder, anxiety disorder etc.<br>Outcome of the STOMP initiative, and other e-modules and training programmes for care staff in the field of ID
2. Modelling	Identify (i) intervention components, and (ii) how intervention components interrelate and relate to surrogate or final health outcomes	Co-development of PEP with stakeholders (through focus groups and co-design event) to identify the targets for the intervention (and their relation to health outcomes), its components (content) and model(s) of delivery
3(a) Operationalization	Describe components of a replicable intervention	Formalising of action plans developed in the co-design event by the PEP Development Group to finalise the content and delivery of the PEP
3(b) Piloting exploratory trials	Describe a feasible trial protocol for comparing the intervention to an appropriate alternative	Instead of a full feasibility study we are now proposing an initial field testing and refinement of the PEP
4. Definitive randomized controlled trial	Compare a fully defined theory-based intervention to an appropriate alternative, using a protocol that is reproducible and adequately controlled in a study with appropriate statistical power	We intend to apply for funding for a future definitive RCT using cluster randomisation methods in community care homes for people with ID with a built-in pilot study
5.Long-term implementation and monitoring	Determine whether the intervention and results can be reliably replicated in uncontrolled settings over the long term	Should be future work after the proposed RCT results become available

For peer review only - http://bmjopen.bmj.com/site/about/guidelines.xhtml

SPIRIT 2013 Checklist: Recommended items to address in a clinical trial protocol and related documents*

Section/item	Item No	Description	Addressed on page number
**Administrative information**			
Title	1	Descriptive title identifying the study design, population, interventions, and, if applicable, trial acronym	1
Trial registration	2a	Trial identifier and registry name. If not yet registered, name of intended registry	Not applicable
	2b	All items from the World Health Organization Trial Registration Data Set	Not applicable
Protocol version	3	Date and version identifier	Not applicable
Funding	4	Sources and types of financial, material, and other support	9
Roles and responsibilities	5a	Names, affiliations, and roles of protocol contributors	1 & 9
	5b	Name and contact information for the trial sponsor	9
	5c	Role of study sponsor and funders, if any, in study design; collection, management, analysis, and interpretation of data; writing of the report; and the decision to submit the report for publication, including whether they will have ultimate authority over any of these activities	10
	5d	Composition, roles, and responsibilities of the coordinating centre, steering committee, endpoint adjudication committee, data management team, and other individuals or groups overseeing the trial, if applicable (see Item 21a for data monitoring committee)	Not applicable See page 7 for project management structure

For peer review only - http://bmjopen.bmj.com/site/about/guidelines.xhtml

For peer review only

Introduction

Background and rationale	6a	Description of research question and justification for undertaking the trial, including summary of relevant studies (published and unpublished) examining benefits and harms for each intervention	2
	6b	Explanation for choice of comparators	Not applicable
Objectives	7	Specific objectives or hypotheses	3
Trial design	8	Description of trial design including type of trial (eg, parallel group, crossover, factorial, single group), allocation ratio, and framework (eg, superiority, equivalence, noninferiority, exploratory)	Not applicable

Methods: Participants, interventions, and outcomes

Study setting	9	Description of study settings (eg, community clinic, academic hospital) and list of countries where data will be collected. Reference to where list of study sites can be obtained	Not applicable
Eligibility criteria	10	Inclusion and exclusion criteria for participants. If applicable, eligibility criteria for study centres and individuals who will perform the interventions (eg, surgeons, psychotherapists)	6
Interventions	11a	Interventions for each group with sufficient detail to allow replication, including how and when they will be administered	Not applicable but see pages 6 for the description of the intervention which will be used in the field trial.
	11b	Criteria for discontinuing or modifying allocated interventions for a given trial participant (eg, drug dose change in response to harms, participant request, or improving/worsening disease)	Not applicable
	11c	Strategies to improve adherence to intervention protocols, and any procedures for monitoring adherence (eg, drug tablet return, laboratory tests)	Not applicable
	11d	Relevant concomitant care and interventions that are permitted or prohibited during the trial	Not applicable

For peer review only - http://bmjopen.bmj.com/site/about/guidelines.xhtml

Outcomes	12	Primary, secondary, and other outcomes, including the specific measurement variable (eg, systolic blood pressure), analysis metric (eg, change from baseline, final value, time to event), method of aggregation (eg, median, proportion), and time point for each outcome. Explanation of the clinical relevance of chosen efficacy and harm outcomes is strongly recommended	Not applicable but see page 6 for care staff related outcome measures
Participant timeline	13	Time schedule of enrolment, interventions (including any run-ins and washouts), assessments, and visits for participants. A schematic diagram is highly recommended (see Figure)	Not applicable but see page 7 for project timeline.
Sample size	14	Estimated number of participants needed to achieve study objectives and how it was determined, including clinical and statistical assumptions supporting any sample size calculations	5
Recruitment	15	Strategies for achieving adequate participant enrolment to reach target sample size	Not applicable but see page 3 for study settings and participants.
**Methods: Assignment of interventions (for controlled trials)**			
Allocation:			
Sequence generation	16a	Method of generating the allocation sequence (eg, computer-generated random numbers), and list of any factors for stratification. To reduce predictability of a random sequence, details of any planned restriction (eg, blocking) should be provided in a separate document that is unavailable to those who enrol participants or assign interventions	Not applicable
Allocation concealment mechanism	16b	Mechanism of implementing the allocation sequence (eg, central telephone; sequentially numbered, opaque, sealed envelopes), describing any steps to conceal the sequence until interventions are assigned	Not applicable
Implementation	16c	Who will generate the allocation sequence, who will enrol participants, and who will assign participants to interventions	Not applicable
Blinding (masking)	17a	Who will be blinded after assignment to interventions (eg, trial participants, care providers, outcome assessors, data analysts), and how	Not applicable

	17b	If blinded, circumstances under which unblinding is permissible, and procedure for revealing a participant's allocated intervention during the trial	Not applicable

Methods: Data collection, management, and analysis

Data collection methods	18a	Plans for assessment and collection of outcome, baseline, and other trial data, including any related processes to promote data quality (eg, duplicate measurements, training of assessors) and a description of study instruments (eg, questionnaires, laboratory tests) along with their reliability and validity, if known. Reference to where data collection forms can be found, if not in the protocol	6: Outcome measure
	18b	Plans to promote participant retention and complete follow-up, including list of any outcome data to be collected for participants who discontinue or deviate from intervention protocols	Not applicable
Data management	19	Plans for data entry, coding, security, and storage, including any related processes to promote data quality (eg, double data entry; range checks for data values). Reference to where details of data management procedures can be found, if not in the protocol	5: Data entry and coding via NVivo & 10 for availability of supporting data
Statistical methods	20a	Statistical methods for analysing primary and secondary outcomes. Reference to where other details of the statistical analysis plan can be found, if not in the protocol	Not applicable, see page 6
	20b	Methods for any additional analyses (eg, subgroup and adjusted analyses)	Not applicable
	20c	Definition of analysis population relating to protocol non-adherence (eg, as randomised analysis), and any statistical methods to handle missing data (eg, multiple imputation)	Not applicable

Methods: Monitoring

Data monitoring	21a	Composition of data monitoring committee (DMC); summary of its role and reporting structure; statement of whether it is independent from the sponsor and competing interests; and reference to where further details about its charter can be found, if not in the protocol. Alternatively, an explanation of why a DMC is not needed	Not applicable as SPECTROM is not an RCT but a combined data management and steering committee will be set up, see page 7

For peer review only - http://bmjopen.bmj.com/site/about/guidelines.xhtml

	21b	Description of any interim analyses and stopping guidelines, including who will have access to these interim results and make the final decision to terminate the trial	Not applicable
Harms	22	Plans for collecting, assessing, reporting, and managing solicited and spontaneously reported adverse events and other unintended effects of trial interventions or trial conduct	Not applicable
Auditing	23	Frequency and procedures for auditing trial conduct, if any, and whether the process will be independent from investigators and the sponsor	Not applicable
**Ethics and dissemination**			
Research ethics approval	24	Plans for seeking research ethics committee/institutional review board (REC/IRB) approval	Not required as no patient data are collected, see page 9
Protocol amendments	25	Plans for communicating important protocol modifications (eg, changes to eligibility criteria, outcomes, analyses) to relevant parties (eg, investigators, REC/IRBs, trial participants, trial registries, journals, regulators)	Not applicable
Consent or assent	26a	Who will obtain informed consent or assent from potential trial participants or authorised surrogates, and how (see Item 32)	9
	26b	Additional consent provisions for collection and use of participant data and biological specimens in ancillary studies, if applicable	Not applicable
Confidentiality	27	How personal information about potential and enrolled participants will be collected, shared, and maintained in order to protect confidentiality before, during, and after the trial	10
Declaration of interests	28	Financial and other competing interests for principal investigators for the overall trial and each study site	9: None declared
Access to data	29	Statement of who will have access to the final trial dataset, and disclosure of contractual agreements that limit such access for investigators	10
Ancillary and post-trial care	30	Provisions, if any, for ancillary and post-trial care, and for compensation to those who suffer harm from trial participation	Not applicable

For peer review only

Dissemination policy	31a	Plans for investigators and sponsor to communicate trial results to participants, healthcare professionals, the public, and other relevant groups (eg, via publication, reporting in results databases, or other data sharing arrangements), including any publication restrictions	8-9
	31b	Authorship eligibility guidelines and any intended use of professional writers	9
	31c	Plans, if any, for granting public access to the full protocol, participant-level dataset, and statistical code	9-10
**Appendices**			
Informed consent materials	32	Model consent form and other related documentation given to participants and authorised surrogates	yes
Biological specimens	33	Plans for collection, laboratory evaluation, and storage of biological specimens for genetic or molecular analysis in the current trial and for future use in ancillary studies, if applicable	No/ Not applicable

*It is strongly recommended that this checklist be read in conjunction with the SPIRIT 2013 Explanation & Elaboration for important clarification on the items. Amendments to the protocol should be tracked and dated. The SPIRIT checklist is copyrighted by the SPIRIT Group under the Creative Commons "Attribution-NonCommercial-NoDerivs 3.0 Unported" license.

For peer review only
