# Supplementary material for: Short-term PsychoEducation for Carers To Reduce Over Medication of people with intellectual disabilities (SPECTROM): study protocol
Source: BMJ Open. 2020 Apr 8;10(4):e037912. doi: 10.1136/bmjopen-2020-037912 (PMC7245413; doi:10.1136/bmjopen-2020-037912)
Supplement: Reviewer comments [file bmjopen-2020-037912.reviewer_comments.pdf]

PEER REVIEW HISTORY

BMJ Open publishes all reviews undertaken for accepted manuscripts. Reviewers are asked to complete a checklist review form (**http://bmjopen.bmj.com/site/about/resources/checklist.pdf**) and are provided with free text boxes to elaborate on their assessment. These free text comments are reproduced below.

ARTICLE DETAILS

**TITLE (PROVISIONAL)**	Short-term PsychoEducation for Carers To Reduce Over Medication of people with intellectual disabilities (SPECTROM): study protocol.
**AUTHORS**	Deb, Shoumitro (Shoumi); Limbu, Bharati; Crawford, Mike; Weaver, Tim

This article was not externally reviewed at BMJ Open. Protocols that have been independently assessed prior to submission to BMJ Open are usually fast-tracked to publication on the grounds that further substantial changes will not be possible. This independent assessment will usually be external, independent review for both a substantial grant award from a non-commercial or government funder and ethics approval.
